# Human Endonuclease ANKLE1 Localizes at the Midbody and Processes Chromatin Bridges to Prevent DNA Damage and cGAS‐STING Activation

**DOI:** 10.1002/advs.202204388

**Published:** 2023-02-24

**Authors:** Huadong Jiang, Nannan Kong, Zeyuan Liu, Stephen C. West, Ying Wai Chan

**Affiliations:** ^1^ School of Biological Sciences The University of Hong Kong Pokfulam Hong Kong; ^2^ The Francis Crick Institute DNA Recombination and Repair Laboratory 1 Midland Road London NW1 1AT UK

**Keywords:** ANKLE1, cGAS‐STING, chromatin bridge, micronucleus, midbody

## Abstract

Chromatin bridges connecting the two segregating daughter nuclei arise from chromosome fusion or unresolved interchromosomal linkage. Persistent chromatin bridges are trapped in the cleavage plane, triggering cytokinesis delay. The trapped bridges occasionally break during cytokinesis, inducing DNA damage and chromosomal rearrangements. Recently, *Caenorhabditis elegans* LEM‐3 and human TREX1 nucleases have been shown to process chromatin bridges. Here, it is shown that ANKLE1 endonuclease, the human ortholog of LEM‐3, accumulates at the bulge‐like structure of the midbody via its N‐terminal ankyrin repeats. Importantly, *ANKLE1^−/−^* knockout cells display an elevated level of G1‐specific 53BP1 nuclear bodies, prolonged activation of the DNA damage response, and replication stress. Increased DNA damage observed in *ANKLE1^−/−^* cells is rescued by inhibiting actin polymerization or reducing actomyosin contractility. ANKLE1 does not act in conjunction with structure‐selective endonucleases, GEN1 and MUS81 in resolving recombination intermediates. Instead, ANKLE1 acts on chromatin bridges by priming TREX1 nucleolytic activity and cleaving bridge DNA to prevent the formation of micronuclei and cytosolic dsDNA that activate the cGAS‐STING pathway. It is therefore proposed that ANKLE1 prevents DNA damage and autoimmunity by cleaving chromatin bridges to avoid catastrophic breakage mediated by actomyosin contractile forces.

## Graphical abstract

Persistent chromatin bridges trapped in the spindle midzone can be broken by actomyosin forces during cytokinesis. This study shows that ANKLE1 is a midbody‐tethered endonuclease that acts directly on DNA bridges trapped in the midbody to prevent the extensive formation of micronuclei and cytosolic DNA. Therefore, ANKLE1 prevents genome instability and the activation of Cyclic GMP‐AMP synthase (cGAS) Stimulator of interferon genes (STING) immune responses.

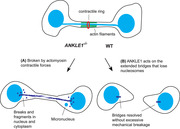

## 1 Introduction

Chromosome segregation defects lead to both structural and numerical chromosomal instability, which is the hallmark of solid tumors.^[^ ^1^ ^]^ Chromosome missegregation can take the form of lagging chromosomes, which are induced by merotelic attachments^[^ ^2^ ^]^ or chromosome breakage that leads to the formation of acentric fragments.^[^ ^3^ ^]^ Missegregation can also be induced by sister chromatid nondisjunction that manifests as chromatin bridges. Chromatin bridges mostly arise from dicentric chromosomes resulting from chromosome end‐to‐end fusions after telomere crisis or erroneous DNA repair,^[^ ^4^ ^]^ or defects in sister chromatid decatenation.^[^ ^5^ ^]^

Persistent chromatin bridges will inevitably be trapped in the midzone during cytokinesis. The presence of DNA bridges within the cleavage plane leads to the activation of Aurora B‐mediated abscission checkpoint in human cells, triggering cytokinesis delay.^[^ ^6^ ^]^ Several consequences of the trapped chromatin have been reported. When a large amount of chromatin is trapped in the cleavage plane, the cleavage furrow would regress and abscission may eventually be aborted, leading to tetraploidization.^[^ ^6c^ ^]^ Alternatively, trapped chromatin can be broken during cytokinesis.^[^ ^6^, ^7^ ^]^ Around 70 years ago, McClintock proposed that chromatin bridges drive chromosome fusions and rearrangements via a so‐called breakage‐fusion‐bridge (BFB) cycle.^[^ ^8^ ^]^ This model predicted that chromatin bridges break apart during cytokinesis and the broken ends subsequently rejoin or rearrange with other broken chromosomes. This model has been supported by multiple observations and experiments. First, frequent BFB events were found in malignant tumors and correlated with the occurrence of anaphase bridges.^[^ ^9^ ^]^ Second, DNA damage was observed in lagging chromosomes near the cleavage furrow.^[^ ^7b^ ^]^ Third, actomyosin ring contraction was shown to break dicentric chromosomes in budding yeast during cytokinesis.^[^ ^10^ ^]^ Similarly, chromatin bridges in human cells were also shown to be broken by actomyosin contractile forces, generating breaks and local DNA fragmentation.^[^ ^7a^ ^]^ Complex rearrangements were then generated following the next defective DNA replication and mitosis. Together, these studies support a model in which trapped chromatin bridges can be mechanically broken by forces of cytokinesis.

However, not all trapped bridges will undergo breakage. It has been shown that chromatin bridges that originate by telomere fusion persist through cytokinesis and develop into long extended bridges connecting the two interphase nuclei.^[^ ^11^ ^]^ The bridges lose nucleosomes, probably due to the forces that result in stretching.^[^ ^12^ ^]^ These extended bridges remain intact for 3–20 h before breakage/resolution. Importantly, the cytoplasmic 3’‐to‐5’ exonuclease TREX1 was shown to accumulate and act on the extended bridges to generate RPA‐coated single‐stranded DNA (ssDNA) after micronuclear envelope rupture during interphase.^[^ ^11^, ^13^ ^]^ These studies provide evidence that cells can utilize specific nucleases to process chromatin bridges to avoid immediate breakage at cytokinesis.

LEM‐3/ANKLE1, a GIY‐YIG domain containing nuclease, has also been implicated in resolving chromatin bridges.^[^ ^14^ ^]^ *Caenorhabditis elegans* LEM‐3 was shown to accumulate at the midbody, a structure where abscission occurs, and colocalize with chromatin bridges trapped at the cleavage plane.^[^ ^15^ ^]^ It was proposed that LEM‐3 resolves DNA bridges that arise from incomplete DNA replication, unresolved recombination intermediates, and defective chromosome condensation at the final stages of cell division. The human ortholog of LEM‐3 is known as ANKLE1.^[^ ^14b^ ^]^ Recombinant ANKLE1 was shown to cleave a range of branched DNA species.^[^ ^16^ ^]^ *ANKLE1* was proposed to be the causal gene in the chr19p13.1 breast and ovarian cancer susceptibility locus,^[^ ^17^ ^]^ suggesting that altered expression of ANKLE1 is involved in tumorigenesis.

Here, we show that the localization of ANKLE1 at the midbody is dependent on its N‐terminal ankyrin repeats and the proper assembly of the central spindle. *ANKLE1^−/−^* knockout cells displayed elevated levels of G1‐specific 53BP1 nuclear bodies and micronuclei. Prolonged activation of the DNA damage response and replication stress were also observed in the following cell cycle upon induction of chromatin bridges. Importantly, increased DNA damage observed in *ANKLE1^−/−^* cells was fully rescued by inhibiting actomyosin contractile ability. Finally, we show that ANKLE1 acts on chromatin bridges to prevent the formation of micronuclei and cytosolic dsDNA fragments that lead to activation of the cGAS‐STING pathway. Together, we propose that the processing of chromatin bridges by ANKLE1 prevents direct mechanical bridge breakage by actomyosin forces, which is more likely to promote fragmentation and catastrophic mutational events in the subsequent cell cycle.

## 2 Results

### 2.1 Human ANKLE1 Localizes to the Midbody

**Figure 1:**
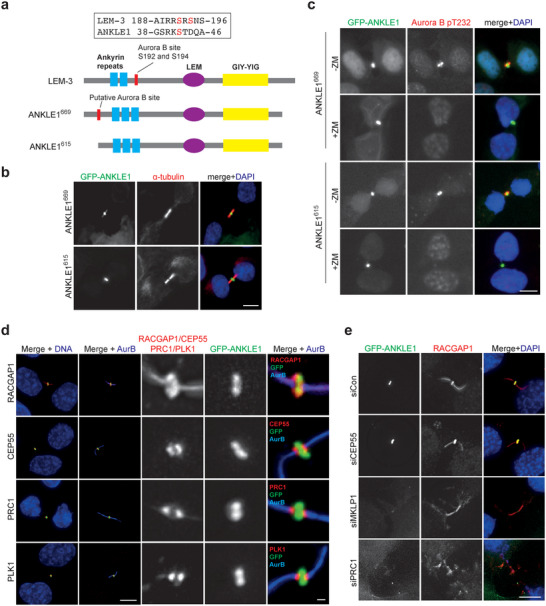
Localization of ANKLE1 at the midbody. a) Schematic representation of the domain structure of LEM‐3 and ANKLE1 (two isoforms: 669 aa and 615 aa). The blue rectangles, purple ellipse, and yellow rectangle represent the ankyrin repeats, LEM domain, and GIY‐YIG nuclease domain, respectively. The putative Aurora B sites are marked by the red rectangles. b) U2OS cells stably expressing GFP‐ANKLE1^669^ and GFP‐ANKLE1^615^ were fixed for immunofluorescence. GFP (green), *α*‐tubulin (red) and DNA (blue) were visualized. c) GFP‐ANKLE1 expressing cells were treated with DMSO or ZM447439 (10 × 10^−6^ m) for 15 min. GFP (green), phosphorylation of Aurora B at T232 (red), and DNA (blue) were visualized. d) GFP‐ANKLE1 expressing cells were fixed for immunofluorescence. GFP (green), different midbody proteins (RACGAP1, CEP55, PRC1 or PLK1 in red) and DNA (blue) were visualized. e) GFP‐ANKLE1 expressing cells were treated with the indicated siRNAs for 48 h before fixed for immunofluorescence. GFP (green), RACGAP1 (red) and DNA (blue) were visualized. Scale bars, 10 µm or 1 µm (inset).

*C. elegans* LEM‐3 was shown to accumulate at the midbody in late mitosis.^[^ ^15b^ ^]^ To study the localization of human ANKLE1, cell lines stably expressing GFP‐ANKLE1 were generated. Two isoforms of ANKLE1 have been reported,^[^ ^16^ ^]^ the 615‐amino‐acid form and the other with an N‐terminal extension of 54 amino acids (**Figure** **1**a). Both isoforms are localized at the midbody (Figure 1b). The 615‐amino‐acid form has been considered as the canonical sequence,^[^ ^14^, ^16^ ^]^ therefore all experiments here were based on this isoform. The localization of ANKLE1 was then investigated in different stages of the cell cycle (Figure S1a, Supporting Information). ANKLE1 showed a diffused localization in early mitosis. It started to accumulate in the spindle midzone in late anaphase and concentrated at the midbody in telophase and cytokinesis. A previous study showed that the midbody localization of LEM‐3 requires the AIR‐2/Aurora B kinase‐mediated phosphorylation at Ser192 and Ser194.^[^ ^15b^ ^]^ A putative Aurora B consensus phosphorylation site (Ser42) was identified in the N‐terminus of the long ANKLE1 isoform (Figure 1a).^[^ ^15b^ ^]^ To test if ANKLE1 localization depends on Aurora B activity, we treated cells with an Aurora B inhibitor, ZM447439,^[^ ^18^ ^]^ which abolished the autophosphorylation of Thr232 of Aurora B at the midbody (Figure 1c).^[^ ^19^ ^]^ However, we found that the localization of both ANKLE1 isoforms was independent of the Aurora B activity as treatment of ZM447439 had no effect on their midbody accumulation (Figure 1c). To test if other mitotic kinases are involved in controlling the midbody recruitment of ANKLE1, we also tested the effect of inhibiting the mitotic kinases PLK1 (by BI2536),^[^ ^20^ ^]^ MPS1 (by reversine),^[^ ^21^ ^]^ or CDK1 (by RO‐3306)^[^ ^22^ ^]^ (Figure S1b, Supporting Information). None of the kinase inhibitors abolished the midbody localization of ANKLE1.

To identify the precise location of ANKLE1 at the midbody, we compared the relative localization of ANKLE1 with other midbody proteins. RACGAP1 and MKLP1 interact and form the centralspindlin complex that plays a critical role in central spindle/midbody assembly.^[^ ^23^ ^]^ ANKLE1 largely colocalized with RACGAP1 in the bulge‐like structure at the center of the midbody (Figure 1d). The microtubule‐bundling proteins PRC1 and CEP55 interact with centralspindlin and are involved in midzone maintenance and ensuing abscission, respectively.^[^ ^24^ ^]^ The staining of PRC1, CEP55, and PLK1 appeared as two closely spaced bands flanking the “dark zone” and overlapped partially with both sides of ANKLE1 staining (Figure 1d). To test if ANKLE1 depends on the midbody proteins to localize to midbody, ANKLE1 staining was examined in cells depleted of MKLP1, CEP55, or PRC1 by siRNAs. Depletion of CEP55 and PRC1 by siRNAs was confirmed by immunofluorescence (IF) staining (Figure S1c,d, Supporting Information). While depletion of MKLP1 was confirmed by the absence of RACGAP1 staining in the midbody (Figure 1e). Importantly, depletion of MKLP1 or PRC1 abolished the midbody localization of both RACGAP1 and ANKLE1, and the midbodies showed clear disorganization (Figure 1e). CEP55 was not required for midbody localization of RACGAP1, nor the integrity of the midbody, and its depletion did not affect ANKLE1 localization (Figure 1e). Together, ANKLE1 midbody localization depends on the formation of the central spindle/midbody mediated by centralspindlin and PRC1.

### 2.2 ANKLE1 Is Required for Proper Cell Division

**Figure 2:**
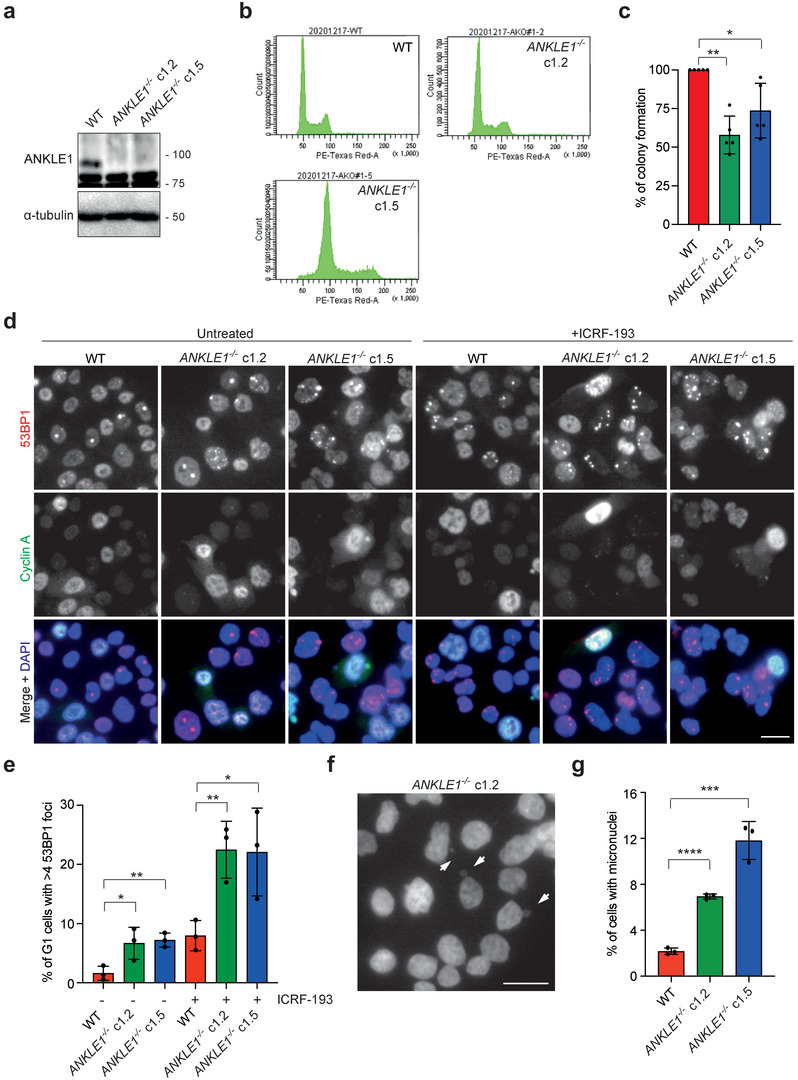
*ANKLE1^−/−^* knockout cells display increased levels of DNA damage and micronuclei. a) Cell extracts of parental HCT116, and two clones of *ANKLE1^−/−^* cells (c1.2 and c1.5) were analyzed by western blotting for the indicated proteins. b) Cell cycle profiles of wild‐type and *ANKLE1^−/−^* cells analyzed by FACS. c) Clonogenic cell survival assay was carried out on wild‐type and *ANKLE1^−/−^* cells. Bars represent mean ± SD of *n* = 5 independent experiments. d) HCT116 wild‐type and *ANKLE1^−/−^* cells were treated with ICRF‐193 (100 × 10^−6^ m) for 16 h before fixed for immunofluorescence. 53BP1 (red), Cyclin A (green) and DNA (blue) were visualized. Scale bar, 10 µm. e) Quantification of G1 cells (>700 cells per condition) with more than four 53BP1 foci. Bars represent mean ± SD of *n* = 3 independent experiments. f) Representative image of *ANKLE1^−/−^* cells, arrows point to micronuclei. Scale bar, 20 µm. g) Quantification of cells with micronuclei (> 700 cells per condition). Bars represent mean ± SD of *n* = 3 independent experiments. *p < 0.05; **p < 0.01; ***p < 0.001; ****p < 0.0001; statistical significance values were determined with unpaired two‐tailed *t*‐tests.

We next sought to determine the cellular functions of endogenous ANKLE1. A previous study suggested that human ANKLE1 is specifically expressed in bone marrow and fetal hematopoietic tissues.^[^ ^14b^ ^]^ However, the expression of ANKLE1 was later found to be less restricted in mice as high expression was also detected in other tissues such as colon, liver, ovary, and testis.^[^ ^25^ ^]^ We first determined whether ANKLE1 was expressed in various non‐transformed and transformed cell lines (hTERT‐RPE1, HCT116, HeLa, U2OS, eHAP and HEK293) by semiquantitative RT‐PCR (Figure S1a–c, Supporting Information). *ANKLE1* mRNA could readily be detected in various cell lines. Next, we generated *ANKLE1^−/−^* knockout cell lines from HCT116 cells using CRISPR (clustered regularly interspaced short palindromic repeats)‐Cas9 technology with a guide RNA targeting exon 4 of *ANKLE1* (Figure S1d, Supporting Information). Six cell clones with abolished expression of ANKLE1 were generated (Figure S2e, Supporting Information). Interestingly, half of them were tetraploids, as indicated by FACS analyses showing their DNA content distribution (Figure S2f, Supporting Information). The high incidence of tetraploidization is in line with the notion that ANKLE1 is involved in safeguarding chromosome segregation as chromosome nondisjunction generates tetraploids.^[^ ^26^ ^]^ The knockout was further verified by DNA sequencing in one diploid and one tetraploid clones, c1.2 and c1.5 (**Figure** **2**a,b and Figure S2d, Supporting Information). *ANKLE1^−/−^* cells displayed a reduced ability to grow into colonies than the wild‐type cells in the clonogenic assay (Figure 2c), indicating that ANKLE1 is required for normal cell proliferation.

### 2.3 ANKLE1 Prevents Chromatin Bridge‐Induced DNA Damage and Genome Instability

If the function of ANKLE1 at the midbody is to process chromatin bridges, unresolved bridges in *ANKLE1^−/−^* cells are expected to be damaged by the forces during cell division, leading to DNA breaks in the next G1 phase. We therefore determined the levels of DNA damage in G1 phase (cyclin A‐negative cells) by measuring the number of 53BP1 nuclear bodies in untreated cells or cells treated with a low dose of topoisomerase II inhibitor, ICRF‐193 (Figure 2d,e). Low‐dose ICRF‐193 treatment is known to induce chromatin bridge formation (Figure S3a,b, Supporting Information).^[^ ^5^ ^]^ As expected, *ANKLE1^−/−^* cells (both c1.2 and c1.5) exhibited a significant increase in the number of G1‐specific 53BP1 nuclear bodies compared with that of the wild‐type cells, in both untreated and ICRF‐193‐treated conditions (Figure 2e). The 53BP1 nuclei bodies represent DNA damage formed by the breakage of chromatin bridges, as the previous report has shown that they were formed following segregation errors and cytokinesis.^[^ ^7b^ ^]^ Consistent with this notion, nearly all 53BP1 foci were observed in untreated *ANKLE1^−/−^* cells colocalized with another well‐known DNA damage marker *γ*H2AX (Figure S3c,d, Supporting Information). We also costained 53BP1 with the centromere, TRF2 (telomeric repeat‐binding factor 2, a telomere marker) and UBF (upstream binding factor, a rDNA‐specific marker), and observed a minor or no colocalization between 53BP1 foci and centromeric/telomeric/rDNA loci in *ANKLE1^−/−^* cells (Figure S3c,d, Supporting Information). Therefore, the DNA damage induced in *ANKLE1^−/−^* cells is not enriched in any particular repetitive elements. Damaged chromosomes are expected to missegregate and form micronuclei more frequently, and indeed *ANKLE1^−/−^* cells exhibited increased micronuclei formation (Figure 2f,g).

**Figure 3:**
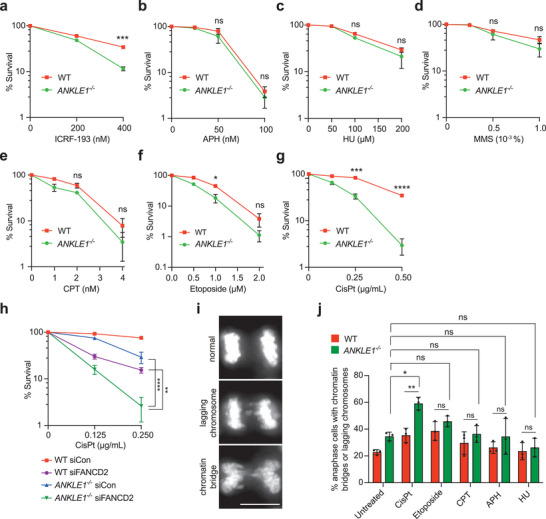
*ANKLE1^−/−^* cells are hypersensitive to cytotoxic drugs that induce chromatin bridges. a–g) Clonogenic cell survival assays were carried out on HCT116 wild‐type and *ANKLE1^−/−^* cells upon treatment of the indicated concentrations of cytotoxic drugs (ICRF‐193, APH: aphidicolin, HU: hydroxyurea, MMS: methyl methanesulfonate, CPT: camptothecin, etoposide and CisPt: cisplatin). Graphs show mean ± SD of *n* = 3 independent experiments. Statistical significance values were determined with unpaired two‐tailed *t*‐tests. h) Clonogenic survival assay was carried out on cells treated with control siRNA or siRNA against FANCD2, followed by the treatment with indicated concentrations of cisplatin. Graphs show mean ± SD of *n* = 3 independent experiments. Statistical significance values were determined with two‐way ANOVA. i) Representative images of anaphase cells harboring lagging chromosome and chromatin bridge. Scale bar, 10 µm. j) Cells were untreated or treated with CisPt (0.5 µg mL^−1^), CPT (10 × 10^−9^ m), APH (200 × 10^−9^ m) or HU (0.5 × 10^−3^ m) for 8 h followed by release into fresh medium for 16 h. Percentages of anaphase cells with lagging chromosome or chromatin bridges were quantified. Bars represent mean ± SD of *n* = 3 independent experiments. Statistical significance values were determined with unpaired two‐tailed *t*‐tests. *p < 0.05; **p < 0.01; ***p < 0.001; ****p < 0.0001; ns = not significant.

To further explore the functional significance of ANKLE1, we tested the sensitivity of *ANKLE1^−/−^* cells to different DNA damaging agents. *ANKLE1^−/−^* cells showed increased sensitivity to low doses of ICRF‐193, but not to cytotoxic agents such as aphidicolin, hydroxyurea, methyl methanesulfonate, camptothecin, etoposide (**Figure** **3**a–f) and nocodazole (Figure S3e, Supporting Information). They were, however, hypersensitive to the DNA crosslinking agent cisplatin (Figure 3g). To rule out the possibility that ANKLE1 is involved in the DNA interstrand crosslink (ICL) repair, wild‐type or *ANKLE1^−/−^* cells were depleted of FANCD2 (Figure S3g, Supporting Information), a core component of the Fanconi anemia pathway that mediates ICL repair.^[^ ^27^ ^]^ FANCD2‐depleted *ANKLE1^−/−^* cells exhibited an exacerbated reduction of cell survival upon cisplatin treatment (Figure 3h), indicating that FANCD2 and ANKLE1 do not act in the same pathway. Similarly, FANCD2‐depleted *ANKLE1^−/−^* cells exhibited a stronger accumulation of G2 cells (with 4N DNA content) (Figure S3h, Supporting Information). The increased sensitivity in *ANKLE1^−/−^* cells upon cisplatin treatment was associated with the extensive formation of chromatin bridges in anaphase, while the other cytotoxic agents did not induce a significant increase in the number of chromatin bridges (Figure 3i,j and Figure S3f, Supporting Information), explaining why ANKLE1 knockout only induces hypersensitivity to cisplatin.

### 2.4 The Ankyrin Repeats and the Nuclease Activity Are Essential for the Function of ANKLE1

**Figure 4:**
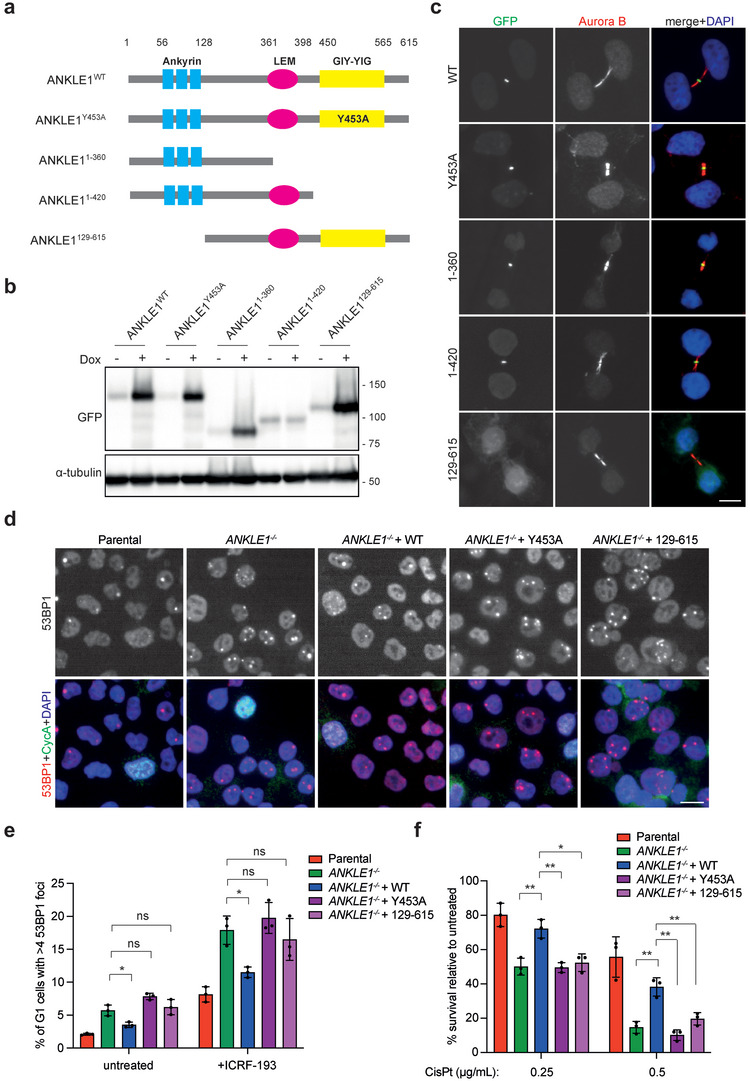
The ankyrin repeats and the nuclease activity are essential for the function of ANKLE1. a) Schematic representation of a series of ANKLE1 truncations and the inactive mutant. b) U2OS cells expressing GFP‐tagged constructs were treated with or without 1 µg mL^−1^ of doxycycline (Dox) that induces protein expression for 24 h. Cell extracts were analyzed by western blotting for the indicated proteins. c) Dox‐treated cells in b) were fixed for immunofluorescence. GFP (green), Aurora B (red) and DNA (blue) were visualized. d) Cells were treated with ICRF‐193 (100 × 10^−9^ m) for 16 h before fixed for immunofluorescence. 53BP1 (red), cyclin A (green), and DNA (blue) were visualized. e) Quantification of G1 cells (>1200 cells per condition) with more than four 53BP1 foci. Bars represent mean ± SD of *n* = 3 independent experiments. f) Clonogenic survival assay was carried out on cells treated with the indicated concentrations of cisplatin. Bars represent mean ± SD of *n* = 3 independent experiments. Scale bars, 10 µm. *p < 0.05; **p < 0.01; ns = not significant; statistical significance values were determined with unpaired two‐tailed *t*‐tests.

The above results are in line with the notion that ANKLE1 is a midbody‐tethered nuclease contributing to DNA bridge processing to prevent induction of DNA damage. To correlate the ANKLE1 midbody localization and bridge resolution, we identified the region of ANKLE1 that is important for its midbody accumulation. A series of truncations fused in‐frame with GFP were generated (**Figure** **4**a,b). The expression of ANKLE1^1‐420^ was much weaker than the others, suggesting that this truncation was less stable. Indeed, treating cells with a proteasome inhibitor MG132 increased the expression level of ANKLE1^1‐420^ (Figure S4a, Supporting Information). We also mutated the Tyr453 residue within the GIY‐YIG nuclease motif, which is crucial for ANKLE1 nuclease activity,^[^ ^14b^ ^]^ to alanine (ANKLE1^Y453A^). The subcellular localization of each expressed protein was determined, and we found that only ANKLE1^129‐615^ failed to localize at the midbody (Figure 4c). These results indicate that the midbody localization of ANKLE1 is dependent on its N‐terminal ankyrin repeats. To confirm this, we generated a cell line expressing GFP‐ANKLE1^1‐128^. This fragment containing only the ankyrin repeats was indeed sufficient to localize at the midbody (Figure S4b,c, Supporting Information). To determine whether the N‐terminal ankyrin repeats and the nuclease activity are essential for the function of ANKLE1, we expressed the ANKLE1^WT^, ANKLE1^129‐615^ or ANKLE1^Y453A^ in *ANKLE1^−/−^* cells (Figure S4d, Supporting Information). Cells were treated with or without a low dose of ICRF‐193 and the number of 53BP1 nuclear bodies in G1 cells was quantified. Expression of ANKLE1^WT^, but not ANKLE1^129‐615^ or ANKLE1^Y453A^, significantly reduced the number of G1‐specific 53BP1 nuclear bodies (Figure 4d,e). Similarly, expression of ANKLE1^129‐615^ or ANKLE1^Y453A^ in *ANKLE1^−/−^* cells could not rescue the viability upon cisplatin treatment (Figure 4f). These results suggest that both the ability to localize at the midbody and the nuclease activity of ANKLE1 are essential for preventing DNA damage induced by chromatin bridges.

### 2.5 ANKLE1‐Mediated Bridge Resolution Prevents Catastrophic Breakage

**Figure 5:**
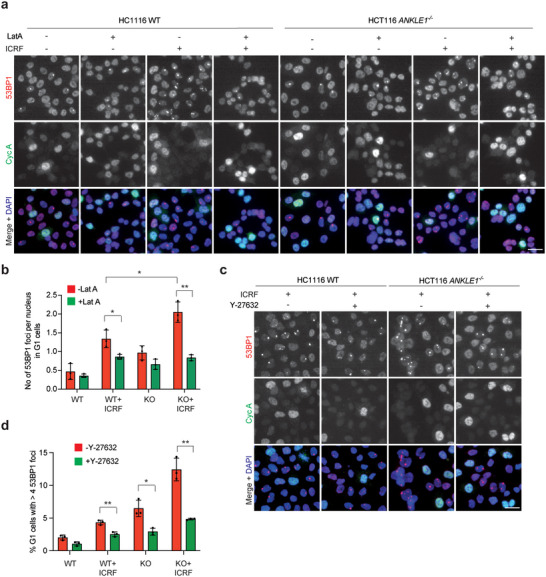
Bridge breakage induced by actomyosin contractile forces in *ANKLE1^−/−^* cells. a) HCT116 wild‐type and *ANKLE1^−/−^* cells were treated with or without ICRF‐193 (100 × 10^−9^ m) for 16 h. Latrunculin A (LatA, 0.5 × 10^−6^ m) was added to cells 6 h before fixation. 53BP1 (red), cyclin A (green) and DNA (blue) were visualized. b) Quantification of 53BP1 foci in G1 cells (>300 cells per condition), treated as in a). c) Wild‐type and *ANKLE1^−/−^* cells were treated with or without ICRF‐193 (100 × 10^−9^  m) and Y‐27632 (20 × 10^−6^ m) for 16 h. 53BP1 (red), Cyclin A (green) and DNA (blue) were visualized. d) Quantification of G1 cells (> 1500 cells per condition), treated as in c), with more than four 53BP1 foci. Bars represent mean ± SD of *n* = 3 independent experiments. *p < 0.05; **p < 0.01; statistical significance values were determined with unpaired two‐tailed *t*‐tests.

A recent study showed that stretched chromatin bridges are broken by the contractile forces of actomyosin.^[^ ^7a^ ^]^ To determine if the G1‐specific 53BP1 damage foci were induced by the breakage of the chromosome by cytokinesis forces, wild‐type or *ANKLE1^−/−^* cells were exposed to an actin polymerization inhibitor, latrunculin A (LatA) that blocks cytokinesis by inducing actin filament depolymerization. We found that *ANKLE1^−/−^* cells exhibited a significant higher number of 53BP1 foci compared with the wild‐type cells upon ICRF‐193 treatment (**Figure** **5**a,b). Importantly, LatA treatment substantially reduced the amount of ICRF‐193‐induced G1‐specific 53BP1 foci in both wild‐type and *ANKLE1^−/−^* cells to a similar basal level (Figure 5a,b). Furthermore, we employed a ROCK (Rho‐associated protein kinase) inhibitor Y‐27632 to reduce actomyosin contractility. Inhibiting ROCK activity has been shown to prevent DNA damage and genome instability.^[^ ^28^ ^]^ Importantly, the number of G1‐specific 53BP1 foci was restored to close to the baseline level when wild type (WT) or *ANKLE1^−/−^* cells were treated with Y‐27632, in both untreated and ICRF‐193‐treated conditions (Figure 5c,d). These results suggest that the action of ANKLE1 in midbody prevents excessive damage of chromosome bridges mediated by actomyosin contractility.

**Figure 6:**
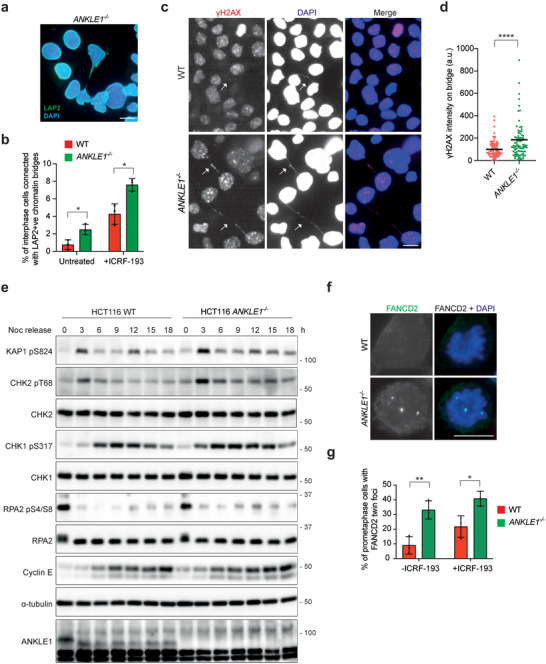
Bridge breakage by actomyosin contractile force induces severer DNA damage in *ANKLE1^−/−^* cells. a) A representative image of an extended chromatin bridge surrounded by LAP2. b) Wild‐type and *ANKLE1^−/−^* cells were treated with or without ICRF‐193 (100 × 10^−9^ m). Quantification of interphase cells (>3000 cells per condition) showing extended bridges surrounded by LAP2. Bars represent mean ± SD of *n* = 3 independent experiments. c) Wild‐type and *ANKLE1^−/−^* cells were treated with ICRF‐193 (100 × 10^−9^ m) for 16 h. *γ*H2AX (red) and DNA (blue) were visualized. d) Quantification of the signal intensity of *γ*H2AX on the extended bridges (*n* = 80 bridges per condition) in cells treated as in c). e) Wild‐type and *ANKLE1^−/−^* cells were shake‐off from overnight nocodazole (100 ng mL^−1^) treatment, followed by release into media with ICRF‐193 (100 × 10^−9^  m) for the indicated time points. Cell extracts were analyzed by western blotting for the indicated proteins. f) Cells were treated with or without ICRF‐193 (100 × 10^−9^ m, 20 h) before arrested in G2 phase by RO‐3306 (9 × 10^−6^ m, 18 h), then released into prometaphase. Cells were fixed and FANCD2 (green) and DNA (blue) were visualized. g) Quantification of prometaphase cells (>150 cells per condition) with FANCD2 twin foci on mitotic chromosomes as in f). Bars represent mean ± SD of *n* = 3 independent experiments. Scale bars, 10 µm. *p < 0.05; **p < 0.01; ****p < 0.001; statistical significance values were determined with unpaired two‐tailed *t*‐tests.

Persistent chromatin bridges were shown to later develop into long extended bridges connecting the two interphase nuclei, and these extended bridges are surrounded by nuclear envelope.^[^ ^11^ ^]^ We therefore measured the number of extended chromatin bridges connecting the two interphase nuclei in wild‐type and *ANKLE1^−/−^* cells. We expect that ANKLE1 processes bridge DNA to prevent the formation of stretched bridges. Indeed, *ANKLE1^−/−^* cells displayed a significant increase in stretched chromatin bridges surrounded by the transmembrane nuclear envelope protein LAP2 between two interphase nuclei (**Figure** **6**a,b). Since bridge breakage by actomyosin contractile forces was shown to generate DNA breaks and fragmentation,^[^ ^7a^ ^]^ one possible explanation of the observed cellular defects in *ANKLE1^−/−^* cells is that, in the absence of ANKLE1, mechanical breakage of chromatin bridges generates more stochastic DNA damage. We quantified the intensity of *γ*H2AX signal on the stretched chromatin bridges. As expected, stretched chromatin bridges induced by ICRF‐193 treatment in *ANKLE1^−/−^* cells exhibited higher intensity of *γ*H2AX staining compared with those of wild‐type cells (Figure 6c,d). To test if DNA damage induced by bridge breakage is more persistent in *ANKLE1^−/−^* cells, nocodazole shake‐off of wild‐type and *ANKLE1^−/−^* cells, followed by release into fresh medium with a low dose of ICRF‐193, was performed and cells were collected at different time points. *ANKLE1^−/−^* cells showed a stronger and more prolonged activation of the DNA damage response (DDR), including phosphorylation of the ATM/ATR targets KAP1, CHK1 and CHK2, and phosphorylation of RPA2 at Ser4/Ser8 by DNA‐PK (Figure 6e). Activation of the DDR persisted even when *ANKLE1^−/−^* cells entered S phase (12 h to 18 h after release from nocodazole, as judged by the high level of cyclin E). This led us to speculate that the breakage of bridges in the absence of ANKLE1 induces replication stress due to the presence of unrepaired damage. To investigate this, cells were first treated with or without a low dose of ICRF‐193, then arrested in the late G2 phase by a CDK1 inhibitor RO‐3306 followed by release into mitosis. A significant higher number of FANCD2 twin foci, which are the marker of replication stress,^[^ ^29^ ^]^ was observed in the prometaphase chromosomes of *ANKLE1^−/−^* cells compared with the wild‐type cells (Figure 6f,g), indicating that *ANKLE1^−/−^* cells experienced replication stress in the subsequent cell cycle after induction of chromatin bridges. Together, these results suggest that ANKLE1 is important to process DNA bridges to prevent bridge breakage solely by mechanical forces that induces more complex lesions in the subsequent cell cycle.

### 2.6 ANKLE1 Does Not Act in Parallel to MUS81 and GEN1 in the Processing of Recombination Intermediates

**Figure 7:**
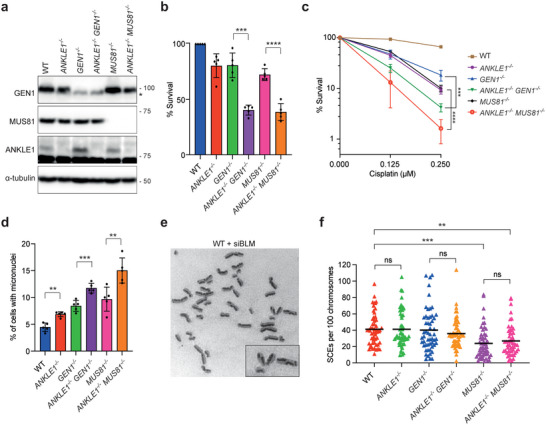
ANKLE1 does not act in parallel to MUS81 and GEN1 in processing recombination intermediates. a) Cell extracts of HCT116 wild‐type, single knockout (*ANKLE1^−/−^*, *GEN1^−/−^* and *MUS81^−/−^*) and double knockout (*ANKLE1^−/−^/GEN1^−/^* ^−^ and *ANKLE1^−/−^/MUS81^−/−^*) cells were analyzed by western blotting for the indicated proteins. The asterisk indicates a cross‐reactive band in GEN1 western blot. b) Clonogenic survival assay was carried out on the indicated cell lines in untreated condition. Bars represent mean ± SD of *n* = 5 independent experiments. Statistical significance values were determined with unpaired two‐tailed *t*‐tests. c) Clonogenic survival assay was carried out on the indicated cell lines upon treatment of the indicated concentrations of cisplatin. Graph shows mean ± SD of *n* = 3 independent experiments. Statistical significance values were determined with two‐way ANOVA. d) Quantification of cells with micronuclei (>3000 cells per condition). Bars represent mean ± SD of *n* = 5 independent experiments. Statistical significance values were determined with unpaired two‐tailed *t*‐tests. e) Representative image of metaphase spread from BLM‐depleted cells showing examples of chromosomes with SCE. f) Quantification of SCE formation in wild‐type, single‐knockout and double‐knockout cells depleted of BLM. At least 50 metaphase cells (>2000 chromosomes) were counted per condition. Statistical significance values were determined with unpaired two‐tailed *t*‐tests. Black lines represent the mean numbers of SCEs per 100 chromosomes per spread. **p < 0.01; ***p < 0.001; ****p < 0.001; ns = not significant.

Recombination intermediates such as Holliday junctions (HJs) are resolved by structure‐selective endonucleases (SSEs).^[^ ^30^ ^]^ These SSEs can be divided into two genetically redundant pathways, conferred by GEN1 and the SMX trinuclease complex composed of MUS81‐EME1, SLX1‐SLX4, and XPF‐ERCC1.^[^ ^31^ ^]^ Recent work in *C. elegans* suggests that LEM‐3 acts in parallel to SLX1‐1/MUS‐81 pathways to process recombination intermediates in both mitotic and meiotic cell division.^[^ ^15^ ^]^ To determine the genetic relationship between ANKLE1 and other SSEs in human cells, we generated single knockout (*GEN1^−/−^* and *MUS81^−/−^*) and double knockout (*ANKLE1^−/−^/GEN1^−/−^* and *ANKLE1^−/−^/MUS81^−/−^*) cell lines (**Figure** **7**a). We found that the double knockout cells showed a significant further reduction in viability compared with single knockout cells (Figure 7b). Moreover, the double knockout cells exhibited a higher cisplatin sensitivity compared with single knockout cells (Figure 7c), demonstrating the synthetic relationship between ANKLE1 and GEN1/MUS81. To determine if the loss of viability in endonuclease‐deficient cells is due to chromosome segregation defects, the formation of micronuclei was quantified. All single knockout cells showed elevated levels of micronuclei compared with wild‐type cells. Importantly, double knockout cells exacerbated the formation of micronuclei (Figure 7d), indicating ANKLE1, MUS81, and GEN1 act independently to prevent chromosome missegregation.

GEN1 and MUS81 resolve replication/recombination intermediates in mitosis to generate sister chromatid exchanges (SCEs).^[^ ^31^, ^32^ ^]^ To investigate whether ANKLE1 acts in parallel to GEN1 and MUS81 in the resolution of DNA intermediates, we compared the SCE levels of wild‐type, single knockout, and double knockouts cells depleted of BLM (Figure 7e,f). Depletion of BLM (which mediates HJ dissolution) leads to an elevated frequency of SCEs resulting from SSEs‐mediated resolution as the resolution pathways are a safeguard mechanism for removal of replication/recombination intermediates that elude dissolution.^[^ ^31b^ ^]^ *MUS81^−/−^* knockout, but not *GEN1^−/−^* knockout, cells displayed a significant reduction of SCE formation, suggesting that MUS81 plays a more prominent role in resolution in HCT116 cells (Figure 7f). ANKLE1 knockout alone did not alter SCE level, and importantly, *ANKLE1^−/−^/MUS81^−/−^* double knockout cells showed no difference in SCE levels to that of *MUS81^−/−^* cells (Figure 7f). These results argue against a possible role for ANKLE1 in the processing of recombination intermediates in parallel with the known resolution pathways. It thus appears that ANKLE1 does not act on recombination intermediates directly but instead processes the chromatin bridges that arise due to unresolved replication/recombination intermediates in GEN1 or MUS81 deficient cells.

### 2.7 ANKLE1 Cleaves Bridge DNA to Prevent the Activation of cGAS‐STING Pathway

**Figure 8:**
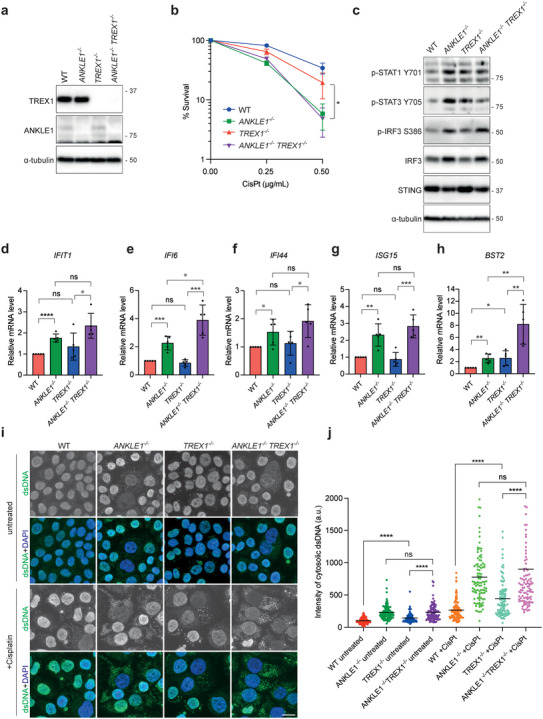
ANKLE1 deficiency induces the activation of the cGAS‐STING pathway. a) Cell extracts of HCT116 wild‐type, *ANKLE1^−/−^, TREX1^−/−^* and *ANKLE1^−/−^/TREX1^−/−^* cells were analyzed by western blotting for the indicated proteins. b) Clonogenic survival assay was carried out on cells treated with the indicated concentrations of cisplatin. Graph shows mean ± SD of *n* = 3 independent experiments. Statistical significance values were determined with two‐way ANOVA. c) Cell extracts were analyzed by western blotting for the indicated proteins. d–h) Relative mRNA levels of the indicated ISGs in cells normalized to untreated HCT116 wild‐type. Bars represent mean ± SD of *n* = 5 independent experiments. Statistical significance values were determined with unpaired two‐tailed *t*‐tests. i) Cells were untreated or treated with cisplatin (0.5 µg mL^−1^, 3 days) and fixed for immunofluorescence. dsDNA (green) and DNA (blue) were visualized. Scale bars, 10 µm. j) Quantification of the signal intensities of the cytoplasmic staining of dsDNA. Intensity of cytosolic dsDNA of a cell is calculated as: the total intensity of dsDNA staining of the whole cell minus the intensity of dsDNA staining in the nucleus. *n* = 100 cells were measured per condition. Statistical significance values were determined with unpaired two‐tailed *t*‐tests. Black lines represent the means in arbitrary unit (a.u.). *p < 0.05; **p < 0.01; ***p < 0.001; ****p < 0.0001; ns = not significant.

We next sought to determine how ANKLE1 acts on DNA bridges. We tested if recombinant ANKLE1 exhibits endonuclease activity on plasmid DNA (Figure S5a, Supporting Information). We found that ANKLE1 cleaves supercoiled plasmids (pcDNA4/TO, 5078 bp) into mostly nicked circular and a small portion of linear products (Figure S5b–e, Supporting Information). ANKLE1 exhibited the same activity to all plasmids tested (pDONR221, pUC19, pSuperior and pEGFP‐C1) (Figure S5f,g, Supporting Information). These results indicate that ANKLE1 generates nicks on plasmids. Double‐strand breaks on the plasmid DNA was also observed at higher enzyme concentrations or with longer reaction time. Previously, the cytoplasmic exonuclease TREX1 was shown to contribute to the resolution of chromatin bridges in interphase by generating extensive ssDNA that is RPA‐coated.^[^ ^11^, ^13^ ^]^ TREX1 requires nicked DNA substrates for generating ssDNA.^[^ ^33^ ^]^ Since ANKLE1 can nick double‐strand DNA, we investigated whether ANKLE1 primes TREX1 nucleolytic activity on bridge DNA. To understand the genetic interaction between ANKLE1 and TREX1, we generated *TREX1^−/−^* single knockout and *ANKLE1^−/−^*/*TREX1^−/−^* double knockout cells (**Figure** **8**a). We identified stretched chromatin bridges between two interphase cells by IF staining of LAP2 and DAPI (Figure S6a,b, Supporting Information). We found that 38% and 8.8% of the chromatin bridges in wild‐type cells and *TREX1^−/−^* cells contained RPA2, respectively, indicating that TREX1 plays a major role in generating ssDNA in chromatin bridges, as previously reported.^[^ ^11^ ^]^ A partial reduction of the appearance of RPA2 on chromatin bridges (24.5%) was observed in *ANKLE1^−/−^* cells. Importantly, *ANKLE1^−/−^*/*TREX1^−/−^* and *TREX1^−/−^* cells showed similar numbers of RPA2‐containing chromatin bridges (10.2% and 8.8%, respectively). These results suggest that ANKLE1 is one of the priming endonucleases for TREX1 in ssDNA generation in chromatin bridges. In line with the notion that TREX1 acts in the same pathway with ANKLE1 in processing chromatin bridges, we also found that the levels of G1‐specific 53BP1 nuclear bodies and micronuclei between *ANKLE1^−/−^* and *ANKLE1^−/−^*/*TREX1^−/−^* cells showed no significant difference (Figure S6c,d, Supporting Information). However, *ANKLE1^−/−^* cells showed a much higher sensitivity to cisplatin than that of *TREX1^−/−^* cells (Figure 8b), suggesting that ANKLE1 plays an additional TREX1‐independent role in resolving bridges, consistent with the biochemical data indicating that ANKLE1 can cleave both strands of DNA (Figure S5, Supporting Information).

Both micronuclei and chromatin bridges are able to activate the innate immunity cGAS‐STING pathway, stimulating the expression of type I interferon (IFN) genes.^[^ ^34^ ^]^ We found that *ANKLE1* knockout led to increased phosphorylation of STAT1, STAT3, and IRF3, which are established markers of cGAS‐STING activation (Figure 8c). To confirm that this is a specific effect, we quantified the signals of IRF3 phosphorylation in the six *ANKLE1^−/−^* knockout cell clones (Figure S6e,f, Supporting Information). All six cell lines displayed significantly increased levels of IRF3 phosphorylation. We also determined the relative mRNA expression levels of a panel of type 1 IFN‐stimulated genes (ISGs: *IFIT1*, *IFI6*, *IFI44*, *ISG15*, and *BST2*) by quantitative RT‐PCR. With the exception of *IFIT1*, we observed a two‐ to fivefold increase in ISG expression in *ANKLE1^−/−^* cells compared with wild‐type cells, in both untreated and cisplatin‐treated conditions (Figure S6g–k, Supporting Information). The expression levels of ISGs in *ANKLE1/TREX1* single/double knockout cells were also determined. *ANKLE1^−/−^*/*TREX1^−/−^* cells showed increased expression of *IFI6* and *BST2* when compared with *ANKLE1^−/−^* cells (Figure 8d–h), suggesting that TREX1 has an additional role in inhibiting cGAS‐STING pathway. For instance, previous studies showed that TREX1 is also involved in degrading cytosolic ssDNA to prevent cGAS activation.^[^ ^35^ ^]^

### 2.8 Micronuclei and Cytosolic dsDNA Activate cGAS‐STING Pathway in *ANKLE1^−/−^* Cells

We expected that one source of the cytosolic immunostimulatory DNA in *ANKLE1^−/−^* cells should be micronuclei. Membrane rupture allows cGAS to bind to micronuclear DNA and micronuclei with ruptured envelopes are often positive for *γ*H2AX.^[^ ^34b^ ^]^ Importantly, *ANKLE1^−/−^* cells displayed not only a significantly higher level of micronuclei when compared to wild‐type cells, but more *γ*H2AX‐positive micronuclei were also observed in both untreated and cisplatin‐treated *ANKLE1^−/−^* cells (Figure S6l–n, Supporting Information). Smaller DNA fragments can also arise from the breakage of chromatin bridges.^[^ ^7^, ^36^ ^]^ Indeed, we observed that deletion of *ANKLE1* promoted the accumulation of cytosolic dsDNA (Figure 8i,j). Treatment of cisplatin led to an even higher level of cytosolic dsDNA in *ANKLE1^−/−^* cells, and *ANKLE1^−/−^*/*TREX1^−/−^* cells did not exhibit a further increase in the accumulation of cytosolic dsDNA when compared with *ANKLE1^−/−^* cells. Such dsDNA is likely to be generated from the damaged bridge DNA.^[^ ^37^ ^]^ We conclude that the extensive formation of chromatin bridges induces both the formation of micronuclei and cytosolic dsDNA in the absence of ANKLE1‐mediated bridge processing, leading to the activation of cGAS‐STING pathway.

To confirm that the induction of ISGs in *ANKLE1^−/−^* cells is due to the activation of cGAS‐STING pathway, we generated *STING^−/−^* and *ANKLE1^−/−^/STING^−/−^* knockout cells (Figure S7a, Supporting Information), and the relative mRNA expression levels of a panel of type 1 ISGs were determined (Figure S7b–e, Supporting Information). Cisplatin treatment induced a four‐ to fivefold increase in ISG expression in *ANKLE1^−/−^* cells compared with wild‐type cells. As expected, the expression levels of ISGs in *ANKLE1^−/−^/STING^−/−^* cells were significantly reduced, to close to the levels observed in *STING^−/−^* cells. These results suggest that the induction of ISGs in *ANKLE1^−/−^* cells depends on the cGAS‐STING pathway.

## 3 Discussion

Chromatin bridges are a potential source of genome instability in cancer cells. It has long been known that Aurora B‐mediated abscission checkpoint stabilizes the ingressed furrow and delays abscission when chromatin is present in the cleavage plane.^[^ ^6^ ^]^ This checkpoint allows more time for bridge resolution and prevents tetraploidization. Unresolved chromatin bridges will inevitably be broken by actomyosin contractile forces, leading to DNA breaks, fragments, and micronuclei formation.^[^ ^7^, ^9^, ^36^, ^38^ ^]^ More complex rearrangements, including chromothripsis and other catastrophic mutational patterns, can be generated when the remnants of broken bridges go to another round of DNA replication and mitosis.^[^ ^7a^ ^]^ Previous studies have shown that *C. elegans* LEM‐3 (the ortholog of human ANKLE1) accumulates at the midbody during the embryonic and meiotic cell cycle to bind and process DNA bridges.^[^ ^15^ ^]^ In this work, we found that, similar to LEM‐3, ANKLE1 also localizes at the midbody in late mitosis and cytokinesis. The N‐terminal ankyrin repeats, which are commonly involved in protein–protein interaction,^[^ ^39^ ^]^ are essential for ANKLE1 to localize to the midbody. Unlike LEM‐3, the midbody localization of ANKLE1 does not depend on Aurora B activity. Since ANKLE1 starts to accumulate in the spindle midzone in late anaphase and its midbody localization depends on centralspindlin and PRC1, we conclude that ANKLE1 interacts with components of the central spindle via its ankyrin repeats and then concentrates at the midbody. The interacting partners of ANKLE1 in the central spindle/midbody remain to be identified. Previously, it was shown that ANKLE1 predominantly localizes to the cytoplasm during interphase driven by a nuclear export signal located in its central region that mediates CRM1‐dependent export.^[^ ^40^ ^]^ This suggests that ANKLE1 is unlikely to act on nuclear DNA in the early stages of the cell cycle.

We found that *ANKLE1^−/−^* cells were hypersensitive to ICRF‐193 and cisplatin. ANKLE1 is not directly involved in DNA damage repair. Instead, *ANKLE1^−/−^* cells are only sensitive to cytotoxic drugs that induce the extensive formation of chromatin bridges. We propose that ANKLE1 processes chromatin bridges trapped in the midzone to prevent excessive DNA damage and micronuclei formation induced by bridge breakage mediated by actomyosin contractile forces. This notion is supported by several results. First, the function of ANKLE1 is dependent on both its ability to localize to the midbody and its nuclease activity. Secondly, blocking cytokinesis by LatA treatment or inhibiting actomyosin contraction by Y‐27632 treatment rescued the induction of DNA damage in *ANKLE1^−/−^* cells. Thirdly, *ANKLE1^−/−^* cells displayed a significant increase in extended bridges between two interphase nuclei. The extended bridges in *ANKLE1^−/−^* cells also displayed higher intensity of *γ*H2AX staining compared with those in wild‐type cells. Finally, increased formation of micronuclei, elevated accumulation of cytosolic dsDNA, and more prolonged activation of the DDR were observed in *ANKLE1^−/−^* cells compared with wild‐type cells upon induction of chromatin bridges. Together, our study suggests that ANKLE1 safeguards genome stability by preventing bridge‐induced DNA damage.

TREX1 exonuclease was recently found to contribute to the resolution of chromatin bridges by acting on nicks in the bridge DNA to generate RPA‐coated ssDNA.^[^ ^11^ ^]^ We showed that recombinant ANKLE1 is capable of nicking double‐stranded plasmids, consistent with a role in generating nicks on chromatin bridges that allow subsequent processing by TREX1. However, ANKLE1 knockout only partially reduced the appearance of RPA‐coated ssDNA on chromatin bridges, suggesting that there are other priming nucleases for TREX1. The nicks in the bridge DNA could also be generated by APE1, NM23‐H1 (both are TREX1‐associated endonucleases) and RHaseH2 (endoribonuclease that mediates the removal of misincorporated ribonucleotides).^[^ ^11^, ^13^ ^]^ Furthermore, ANKLE1 may be able to process bridges independent of TREX1 as *ANKLE1^−/−^* cells displayed a much higher cisplatin sensitivity than *TREX1^−/−^* cells. Since ANKLE1 also cleaves both strands of DNA in vitro, it is possible that the stretching of chromatin bridges results in nucleosome loss and exposes the naked dsDNA for ANKLE1‐mediated cleavage and resolution.

In addition, we observed that cGAS‐STING pathway was activated in *ANKLE1^−/−^* cells. cGAS is an important cytosolic dsDNA sensor that induces type I IFN response via its adaptor STING.^[^ ^41^ ^]^ We identified two sources of cytosolic dsDNA that are able to induce cGAS‐STING activation in *ANKLE1^−/−^* cells. First, *ANKLE1^−/−^* cells displayed increased micronuclei formation, and it has been shown that micronuclei recruit and activate cGAS upon rupture of their membranes.^[^ ^34^, ^42^ ^]^ Secondly, an accumulation of cytosolic short dsDNA fragments in *ANKLE1^−/−^* cells was observed. Considering the role of ANKLE1 in processing chromatin bridges, we assume that the cytosolic dsDNA is derived from the fragmentation of chromatin bridges by actomyosin contractile forces in *ANKLE1^−/−^* cells. Therefore, we propose that the loss of ANKLE1 induces cGAS‐STING pathway‐mediated immunosurveillance.

Figure S8 (Supporting Information) shows a schematic depicting the cellular consequences when chromatin bridges are trapped at the cleavage plane in wild‐type and *ANKLE1^−/−^* cells. Chromatin bridge stretching, by the pulling force of migrating daughter cells, leads to loss of nucleosomes and exposure of naked DNA.^[^ ^43^ ^]^ In wild‐type cells, ANKLE1 at the midbody acts on the extended bridges in two possible ways: ANKLE1 nicks bridge DNA to prime TREX1 nucleolytic activity to generate ssDNA for further resolution. ANKLE1 can also directly resolve bridges by cleaving both strands of bridge DNA. ANKLE1's actions generate relatively less amount of DNA damage that will be repaired before the S phase. In *ANKLE1^−/−^* cells, chromatin bridges will eventually be broken by the actomyosin contractile forces during cytokinesis in the absence of ANKLE1‐mediated processing. Bridge breakage is random, leading to nuclear DNA damage, micronuclei, and cytosolic dsDNA formation. Both micronuclei and DNA damage can lead to defective replication that induces additional damage and rearrangements in the subsequent cell cycle. Furthermore, micronuclei and small fragments of DNA in the cytoplasm generated by bridge breakage are detected by the cytosolic dsDNA sensor cGAS to activate type I interferon response via its adaptor STING, inhibiting cell growth. Hence, our work suggests that ANKLE1 serves as a final safeguard system to resolve trapped chromatin bridges before abscission, preventing stochastic breakage of bridge DNA by mechanical forces, which leads to genome instability and innate immune responses.

## 4 Experimental Section

### Plasmids

Codons optimized ANKLE1^615^ (for expression in *Escherichia coli*) carrying an N‐terminal 6xHis tag cloned in pET100/D‐TOPO vector was purchased from GeneArt (Thermo Fisher Scientific). The ANKLE1^669^ cDNA cloned in pcDNA3.1+/N‐eGFP vector was purchased from Genescript (clone ID: OHU12449). The ANKLE1^615^, ANKLE1^129‐615^, ANKLE1^1‐420^, ANKLE1^1‐360^ and ANKLE1^1‐128^ carrying a N‐terminal GFP tag were generated by PCR and cloned into pcDNA5/FRT/TO vector or pcDNA3.1+ vector. The catalytic‐dead mutant of ANKLE1, ANKLE1^Y453A^, was generated using a QuikChange Lightning Multi Site‐Directed Mutagenesis kit (Agilent). To generate the sgRNA vectors for gene knockout, pairs of annealed oligonucleotides were cloned into the pX459 plasmid (Addgene 62988) according to the published protocol.^[^ ^44^ ^]^ The following sequences of sgRNA oligonucleotides were used for gene targeting:

*ANKLE1*: 5’‐CACCGTCCGCGTGTCGAGATCCTGC‐3’ and 5’‐AAACGCAGGATCTCGACACGCGGAC‐3’

*GEN1*: 5’‐CACCGCACATCCCCTTGCGTAATCT‐3’ and 5’‐AAACAGATTACGCAAGGGGATGTGC‐3’^[^ ^45^ ^]^

*MUS81*: 5’‐CACCGTCTGAAATACGAAGCGCGTG‐3’ and 5’‐AAACCACGCGCTTCGTATTTCAGAC‐3’^[^ ^45^ ^]^

*TREX1*: 5’‐CACCGGAGCCCCCCCACCTCTC‐3’ and 5’‐AAACGAGAGGTGGGGGGGCTCC‐3’^[^ ^11^ ^]^

*STING*: 5’‐CACCGCATATTACATCGGATATCTG‐3’ and 5’‐AAACCAGATATCCGATGTAATATGC‐3’

### Protein Purification

Plasmids expressing 6xHis‐ANKLE1^615^ were transformed into One Shot BL21 Star DE3 *E. coli* competent cells (Thermo Fisher Scientific). The BL21 cells were first transformed with the pGro7 plasmids (TaKaRa) expressing groES‐groEL chaperone proteins that increase the recovery of expressed ANKLE1 in the soluble fraction. Transformed BL21 cells were grown in LB medium at 37 °C. L‐Arabinose (0.5 mg mL^−1^) was added to the culture to induce the expression of the chaperone for 30 min when the OD_650_ of the culture reached ≈0.5. The expression of the ANKLE1 from a T7 promoter was induced by the addition of 0.5 × 10^−3^ m IPTG to the culture at OD_650_. ≈0.8 for 6 h at 25 °C. Cells were harvested and resuspended in lysis buffer (40 × 10^−3^ m Tris–HCl pH 7.5, 500 × 10^−3^ m NaCl, 10% glycerol, 0.05% NP40, 1 × 10^−3^ m DTT and protease inhibitor tablets (Roche)). The lysate was then disrupted in a high‐pressure homogenizer (Avestin Emulsiflex C5). The lysate was cleared by ultracentrifugation at 27 000 rpm using a Ti‐70 rotor (Beckman Coulter) for 30 min before incubated with Ni‐NTA agarose beads (Qiagen) for 1.5 h at 4 °C. The beads were washed extensively several times in wash buffer (40 × 10^−3^ m Tris‐HCl pH 7.5, 500 × 10^−3^ m NaCl, 10% glycerol, 1 × 10^−3^ m DTT and 20 × 10^−3^ m imidazole) and once in ATP wash buffer (40 × 10^−3^ m Tris‐HCl pH 7.5, 500 × 10^−3^ m NaCl, 10% glycerol, 1 × 10^−3^ m DTT, 1 × 10^−3^ m ATP, and 3 × 10^−3^ m MgCl_2_). Proteins were eluted with His elution buffer (40 × 10^−3^ m Tris–HCl pH 7.5, 500 × 10^−3^ m NaCl, 10% glycerol, 1 × 10^−3^ m DTT, 250 × 10^−3^ m imidazole). The elution (≈2 mL) was diluted to 100 × 10^−3^ m NaCl and the diluted protein solution was loaded on a Hitrap Q HP anion exchange chromatography column (1 mL) using a AKTA pure system (GE Healthcare). ANKLE1 proteins were then eluted from the column with an increased concentration of NaCl (100 × 10^−3^ m to 1m) in 20 column volumes. The peak fraction was then applied to a Superdex 200 size exclusion chromatography column using a AKTA pure system. ANKLE1 was eluted with the protein storage buffer (40 × 10^−3^ m Tris‐HCl pH 7.5, 500 × 10^−3^ m NaCl, 10% glycerol, 1 × 10^−3^ m DTT). The eluted fractions were analyzed by SDS‐PAGE and Coomassie blue staining to identify the fractions with pure ANKLE1 proteins. Protein concentration of the peak fraction was measured by Bradford protein assay (Bio‐Rad), and the proteins were aliquoted, frozen in liquid nitrogen and stored in ‐80 °C freezer.

### Nuclease Assay

Different plasmids were incubated with ANKLE1 in cleavage buffer (50 × 10^−3^ m Tris‐HCl pH 8.0, 1 × 10^−3^ m MnCl_2_, 1 × 10^−3^ m DTT) at 37 °C. DNA products were deproteinized by the addition of 2.5 µL of stop buffer (100 × 10^−3^ m Tris‐HCl pH 7.5, 50 × 10^−3^ m EDTA, 2.5% SDS and 10 mg mL^−1^ proteinase K) and incubation for 60 min at 37 °C. The products were analyzed by 0.8% agarose gel electrophoresis running in 1× TBE buffer (90 × 10^−3^ m Tris base, 90 × 10^−3^ m boric acid, and 2 × 10^−3^ m EDTA), stained with SYBR Gold (Thermo Fisher Scientific) and imaged with a Gel Doc 2000 System (Bio‐Rad). Reaction products were quantified using Image Lab software.

### Cell Culture and Stable Cell Line Generation

U2OS Flp‐In T‐REx cell line was a gift from Erich Nigg (University of Basel), HCT116 cell line was obtained from ATCC. They are cultured in DMEM medium (Thermo Fisher Scientific) supplemented with 10% fetal bovine serum (cat no. 10270106, Thermo Fisher Scientific) and penicillin–streptomycin (100 U mL^−1^, Thermo Fisher Scientific) at 37 °C in 5% CO_2_. Geneticin (500 µg mL^−1^), hygromycin (100 µg mL^−1^), blasticidin (5 µg mL^−1^), and puromycin (0.5 µg mL^−1^) were obtained from Thermo Fisher Scientific. Nocodazole, ICRF‐193, aphidicolin, hydroxyurea, methyl methanesulfonate, camptothecin, and MG132 (10 × 10^−6^ m) were obtained from Sigma‐Aldrich. ZM447439 (10 × 10^−6^ m) and RO‐3306 (9 × 10^−6^ m) were obtained from Selleckchem. Reversine (0.5 × 10^−6^ m), BI‐2536 (100 × 10^−9^ m) and latrunculin A (0.5 × 10^−6^ m) were obtained from Cayman.

To generate stable U2OS cell lines expressing different constructs of ANKLE1 proteins, U2OS Flp‐In T‐Rex cells were cotransfected with the pcDNA5/FRT/TO plasmids encoding the protein of interest and pOG44 plasmids that encode Flp recombinase (1:9 ratio). Hygromycin‐resistant colonies were picked and expanded. Protein expression was induced by adding 1 µg mL^−1^ doxycycline (Sigma‐Aldrich). To generate stable HCT116 cell lines expressing different constructs of ANKLE1 proteins, HCT116 cells were transfected with pcDNA3.1+ plasmids encoding the protein of interest, and geneticin‐resistant colonies were picked and expanded.

### Generation of Knockout Cell Lines

For gene targeting, HCT116 cells were transfected with pX459 carrying the targeting sequences using lipofectamine 2000 (Thermo Fisher Scientific). After 48 h, the transfected cells were selected with puromycin (0.5 µg mL^−1^) for 48 h, then seeded as single colonies and grown in the medium without puromycin. Clones were picked ≈2 weeks later and expanded. Knockouts were first verified by western blotting. The genomic DNA of the selected knockouts was extracted with a DNeasy Blood & Tissue Kit (Qiagen), and the targeting loci were amplified with the Q5 High‐Fidelity DNA polymerase (New England Biolabs) using the following primers:

*ANKLE1*: 5’‐ AGAGGGAGGGAAGGAAGGTAAG‐3’ and 5’‐ GTCAGGGACTGGTCCAGAAGT‐3’

*GEN1*: 5’‐CTGGCTTATAATATATTGTTTG‐3’ and 5’‐GCTTTTAGTATCTGAAGCATC‐3’^[^ ^45^ ^]^

*MUS81*: 5’‐GAATCCCGACTCCAGAACTG‐3’ and 5’‐GCTCGTCCAGCATCCGGCAG‐3’^[^ ^45^ ^]^

*TREX1*: 5’‐ACATGGAGGCCACTGGCTTG‐3’ and 5’‐CGAGTGTAGATGCTGCCTAG‐3’

The PCR products were purified using a Qiaquick PCR purification kit (Qiagen) and cloned into the pJET vector using a CloneJET PCR cloning kit (Thermo Fisher Scientific). The plasmids were then sequenced to confirm the gene disruption.

### siRNA

siRNA transfection was performed using Lipofectamine RNAiMAX (Thermo Fisher Scientific). Cells were seeded one day before siRNA treatment and transfected with 25 × 10^−9^ m of siRNA. The following siRNAs were used:

Control siRNA: 5’‐UAAUGUAUUGGAACGCAUA‐3’^[^ ^45^ ^]^

CEP55 siRNA: 5’‐GGAGAAGAAUGCUUAUCAA‐3’^[^ ^46^ ^]^

MKLP1 siRNA: 5’‐GAGUGUUGCAUAGAAGUGA‐3’^[^ ^47^ ^]^

PRC1 siRNA: 5’‐AUAUGGGAGCUAAUUGGGA‐3’^[^ ^48^ ^]^

FANCD2 siRNA: 5’‐CAGCCAUGGAUACAUUGATT‐3’

BLM siRNA: 5’‐CCGAAUCUCAAUGUACAUAGA‐3’^[^ ^45^ ^]^

### RT‐PCR

Total RNA was isolated from cells using the TRIzol Reagent (Thermo Fisher Scientific) according to the manufacturer's instructions. The same amount of RNA was reverse transcribed using a High‐Capacity cDNA Reverse Transcription Kit (Thermo Fisher Scientific). Semiquantitative RT‐PCR was performed using the Q5 High‐Fidelity DNA polymerase (New England Biolabs) and the products were analyzed by 1% agarose gel electrophoresis. Quantitative RT‐PCR was performed on a Bio‐Rad CFX96 Touch Real‐Time PCR Detection System (Bio‐Rad) with the TB Green Premix Taq II (TaKaRa). GAPDH was used as the internal control. The relative gene expression was calculated using the 2^−ΔΔCt^ method. The following oligonucleotides were used:

ANKLE1:^[^ ^14b^ ^]^ P1 Forward: 5’‐ TGCCTGTGGGAGCACCAGACATC‐3’,

P2 Forward: 5’‐ GCCCTGCGGACGGGCTGTATTC‐3’,

P3 Reverse: 5’‐GCTCGCCTTCAGCCAGGAAGAC‐3’;

GAPDH:^[^ ^14b^ ^]^ Forward: 5’‐CATCACCATCTTCCAGGAGCGA‐3’,

Reverse: 5’‐CCTGCTTCACCACCTTCTTGAT‐3’;

IFIT1:^[^ ^35a^ ^]^ Forward: 5’‐TACCTGGACAAGGTGGAGAA‐3’,

Reverse: 5’‐GTGAGGACATGTTGGCTAGA‐3’;

IFIT44:^[^ ^35a^ ^]^ Forward: 5’‐ATGGCAGTGACAACTCGTTTG‐3’,

Reverse: 5’‐TCCTGGTAACTCTCTTCTGCATA‐3’;

ISG15:^[^ ^35a^ ^]^ Forward: 5’‐GCGAACTCATCTTTGCCAGTA‐3’,

Reverse: 5’‐CCAGCATCTTCACCGTCAG‐3’;

IFI6:^[^ ^35a^ ^]^ Forward: 5’‐TCGCTGATGAGCTGGTCTGC‐3’,

Reverse: 5’‐ATTACCTATGACGACGCTGC‐3’;

BST2:^[^ ^35a^ ^]^ Forward: 5’‐CCGTCCTGCTCGGCTTT‐3’,

Reverse: 5’‐CCGCTCAGAACTGATGAGATCA‐3’;

### Cell Extracts and Western Blotting

Cell lysates were prepared by resuspending cells in Tris‐lysis buffer (50 × 10^−3^ m Tris‐HCl pH 7.5, 150 × 10^−3^ m NaCl, 0.5% NP40, 1 × 10^−3^ m EDTA, 1 × 10^−3^ m DTT) supplemented with protease inhibitors. The lysates were incubated on ice for 30 min and then cleared by centrifugation (14 000 rpm for 30 min at 4 °C). For western blotting of ANKLE1, RIPA buffer (50 × 10^−3^ m Tris‐HCl pH 7.5, 150 × 10^−3^ m NaCl, 1% Triton X‐100, 0.5% sodium deoxycholate, 0.1% SDS and 5 × 10^−3^ m EDTA) was used to lyse the cells. Protein concentrations were determined using Bradford Assay and equal amounts of total proteins were loaded in each lane of the SDS‐PAGE. Proteins were transferred to nitrocellulose membranes. After blocking with 5% non‐fat milk in PBST, membranes were incubated with primary and secondary antibodies sequentially. Proteins were detected by SuperSignal West Pico Chemiluminescent Substrate (Thermo Fisher Scientific) and the Uvitec Alliance Q9 Mini imaging system. The western blot signals were measured using FIJI software.

### Immunofluorescence

Cells were grown on coverslips and fixed with PTEMF buffer (20 × 10^−3^ m PIPES pH 6.8, 0.2% Triton X‐100, 1 × 10^−3^ m MgCl_2_, 10 × 10^−3^ m EGTA and 4% paraformaldehyde) for 10 min. For staining dsDNA, cells were fixed with 4% paraformaldehyde in PBS for 10 min. Fixed cells were blocked with 3% BSA in PBS for 30 min and then incubated with primary antibodies diluted in 3% BSA in PBS for 1 h, washed with PBS, and incubated with secondary antibodies diluted in 3% BSA in PBS for 1 h. DNA was stained with DAPI. The coverslips were washed twice with PBS and then mounted with Prolong Diamond antifade mountant (Thermo Fisher Scientific) on microscope slides. Images were acquired using a Nikon Ti60 microscope equipped with DS‐Ri2 camera under 40× objective, or a DeltaVision Ultra microscope (Cytiva Life Sciences) equipped with a PlanApo 60×/1.50 oil immersion objective and a CoolSNAP HQ camera (Photomertrics). DeltaVision images at single focal planes were processed with a deconvolution algorithm, and optical sections were projected using maximum intensity projection into one picture using SoftwoRx. Images were processed using Adobe Photoshop. The intensity of immunofluorescence staining was measured using FIJI software.

### Antibodies

Proteins were detected by western blotting or immunofluorescence using the following primary antibodies: rabbit anti‐ANKLE1 (1:500, raised against full length ANKLE1 purified in denatured condition), rabbit anti‐GFP (1:5000, Abcam ab290), mouse anti‐*α*‐tubulin (1:5000, Sigma 00020911), rabbit anti‐Aurora B (1:2000, Abcam ab2254), rabbit anti‐Aurora B phosphor‐T232 (1:1000, Rockland 600‐401‐677), mouse anti‐CEP55 (1:500, Santa Cruz Biotechnology sc‐374051), mouse anti‐PRC1 (1:500, Santa Cruz Biotechnology sc‐376983), mouse anti‐PLK1 (1:1000, Santa Cruz Biotechnology sc‐17783), goat anti‐RACGAP1 (1:400, Abcam ab2270), rabbit anti‐53BP1 (1:1000, Abcam ab36823), mouse anti‐cyclin A (1:200, Santa Cruz Biotechnology sc‐271682), rabbit anti‐KAP1 phospho‐S824 (1:1000, Abcam ab70369), rabbit anti‐CHK2 phospho‐T68 (1:1000, Cell Signaling 2661), rabbit anti‐CHK1 phospho‐S317 (1:1000, Cell Signaling 2344), mouse anti‐CHK2 (1:1000, Millipore 05‐649), mouse anti‐CHK1 (1:1000, Sigma C9358), rabbit anti‐RPA2 phospho‐S4/S8 (1:1000, Bethyl A300‐245A), mouse anti‐RPA2 (1:1000, Abcam ab2175), mouse anti‐cyclin E (1:1000, Cell Signaling 4129), rabbit anti‐FANCD2 (1:1000, Novus NB100‐182), rabbit anti‐GEN1 (1:500, raised against GEN1^890‐908^),^[^ ^49^ ^]^ mouse anti‐MUS81 (1:1000, Santa Cruz Biotechnology sc‐47692), rabbit anti‐TREX1 (1:1000, Abcam ab185228), mouse anti‐LAP2 (1:2000, BD Biosciences 611000), rabbit anti‐pIRF3‐S386 (1:1000, Abcam ab76493), rabbit anti‐IRF3 (1:1000, Abcam ab68481), rabbit anti‐STING (1:1000, Abcam ab181125), rabbit anti‐STAT1 phospho‐Y701 (1:1000, Cell Signaling 9167), rabbit anti‐STAT3 phospho‐Y705 (1:2000, Cell Signaling 9145), mouse anti‐*γ*H2AX (1:1000, Millipore 05‐636), human anti‐centromere CREST (1:2000, Immunovision HCT‐0100), rabbit anti‐TRF2 (1:1000, Novus NB110‐57130), mouse anti‐UBF (1:200, Santa Cruz Biotechnology sc‐13125) and mouse anti‐dsDNA (1:1000, Abcam ab27156). For western blotting, primary antibody detection was performed using HRP‐conjugated goat anti‐mouse or anti‐rabbit antibodies (1:2000, Bio‐Rad 1706515 and 1706516). For immunofluorescence, primary antibody detection was performed using secondary antibodies conjugated to Alexa Fluor 488, Alexa Fluor 546, Alexa Fluor 555 and Alexa Fluor 647 against rabbit, mouse or goat immunoglobulin heavy and light chain (1:2000; Thermo Fisher Scientific).

### Flow Cytometry

Cells were harvested and washed with PBS before fixed in ice‐cold 70% ethanol for 1 h at 4 °C. To analyze DNA content, fixed cells were washed twice with PBS and incubated with 50 µL of RNase A (100 µg mL^−1^) and 400 µL of propidium (50 µg mL^−1^) for 30 min before analyzed by a FACSAria III Cell Sorter (BD Biosciences). At least 10000 cells were acquired per sample. Cell doublets and debris were excluded from the analyses.

### Clonogenic Cell Survival Assay

200 cells were first seeded in six‐well plates. 24 h later, different concentrations of cytotoxic drugs were added. Cells were grown for ≈10 d to allow colony formation. Colonies were stained for ≈2 min with 40 mg mL^−1^ crystal violet solution (Sigma‐Aldrich) containing 20% ethanol.

### Sister Chromatid Exchange Assay

Cells were transfected with BLM siRNA to induce SCEs. 24 h later, BrdU (100 × 10^−6^ m) was added for 48 h, and colcemid (0.2 µg mL^−1^) was added 1 h prior to fixation. The SCE assay was performed as described previously.^[^ ^31b^ ^]^

### Statistical Analysis

Sample sizes were determined based on previous experience to obtain statistical significance and reproducibility. All error bars represent mean ± standard deviation (SD) of *n* = at least 3 independent experiments, unless otherwise specified. Unpaired two‐tailed *t*‐test or two‐way ANOVA were performed using GraphPad Prism 9 software. A *p* value of less than 0.05 was considered statistically significant. The *p* values were indicated above the graphs: *p < 0.05; **p < 0.01; ***p < 0.001; ****p < 0.0001, ns = not significant.

## Conflict of Interest

The authors declare no conflict of interest.

## Author Contributions

H.J. conducted most of the experiments and analyzed the results. N.K. conducted the biochemical experiments. Z.L. assisted the cell line generation. S.C.W. provided crucial reagents and participated in the manuscript preparation. Y.W.C. conceived the project and prepared the manuscript.

## Supporting information

Supporting Information

## Data Availability

The data that support the findings of this study are available from the corresponding author upon reasonable request.
