# Supplementary material for: Human Endonuclease ANKLE1 Localizes at the Midbody and Processes Chromatin Bridges to Prevent DNA Damage and cGAS‐STING Activation
Source: Adv Sci (Weinh). 2023 Feb 24;10(12):2204388. doi: 10.1002/advs.202204388 (PMC10131833; doi:10.1002/advs.202204388)
Supplement: Supplementary file 1 — Supporting Information [file ADVS-10-2204388-s001.pdf]

ADVANCED SCIENCE
Open Access

# Supporting Information

for *Adv. Sci.*, DOI 10.1002/advs.202204388

## Human Endonuclease ANKLE1 Localizes at the Midbody and Processes Chromatin Bridges to Prevent DNA Damage and cGAS-STING Activation

*Huadong Jiang, Nannan Kong, Zeyuan Liu, Stephen C. West and Ying Wai Chan**

**Supporting Information**

# Human Endonuclease ANKLE1 Localizes at The Midbody and Processes Chromatin Bridges to Prevent DNA Damage and cGAS-STING Activation

*Huadong Jiang, Nannan Kong, Zeyuan Liu, Stephen C. West and Ying Wai Chan[*]*

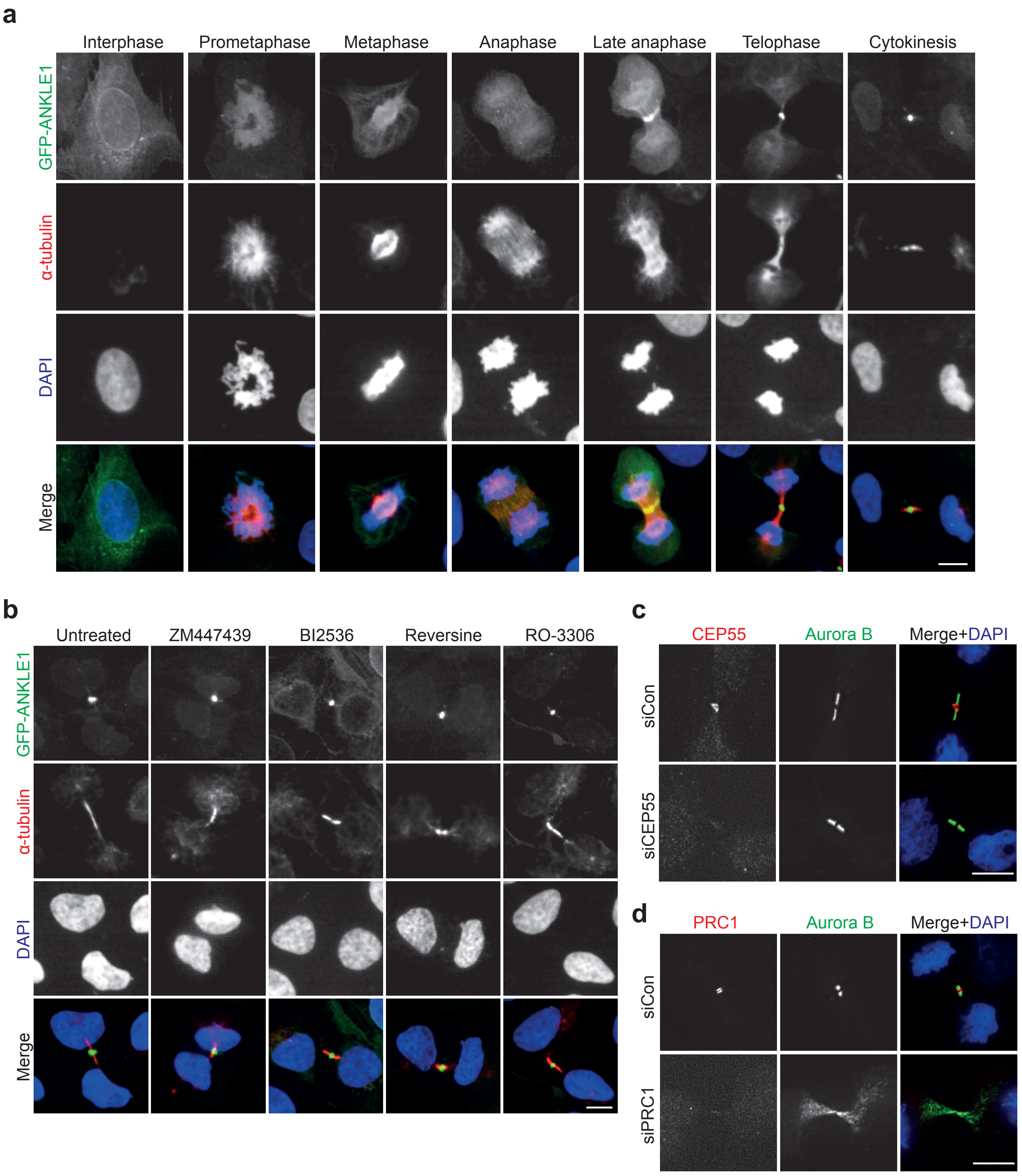


**Figure S1**. Localization of ANKLE1 at the midbody. a) Cells expressing GFP-ANKLE1 were fixed for immunofluorescence. Cells in different stages of cell division were imaged. b) Cells were treated with the indicated kinase inhibitors for 30 min before fixed for immunofluorescence. Telophase cells were imaged in each condition for the localization of ANKLE1. c) Cells were treated with control siRNA or siRNA against CEP55 for immunofluorescence. d) Cells were treated with control siRNA or siRNA against PRC1 for immunofluorescence. Scale bars, 10 μm.

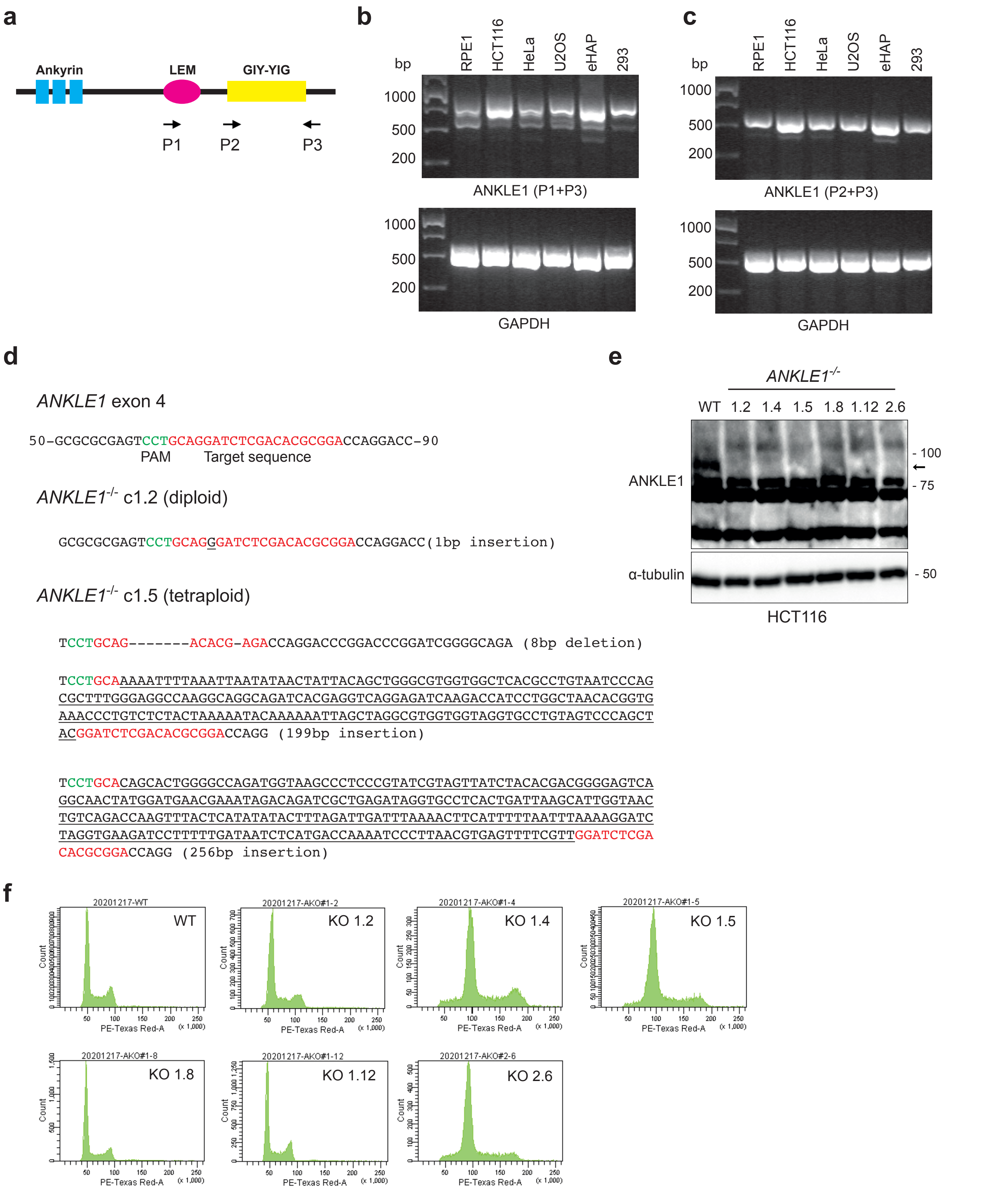


**Figure S2**. Generation of *ANKLE1*$^{-/-}$ knockout HCT116 cells by CRISPR/Cas9. a) Position of P1, P2 and P3 primers for RT-PCR. b) Detection of ANKLE1 transcripts by P1-P3 in different human cell lines. GAPDH was used as controls for equal loading. c) Detection of ANKLE1 transcripts by P2-P3 in different human cell lines. GAPDH was used as control for equal loading. d) The sequence of the sgRNA used to generate ANKLE1 knockout from HCT116 cells. Insertions/deletions in ANKLE1 exon 4 of the two clones (c1.2 and c1.5) are shown. e) Western blot analysis of six individual clones of *ANKLE1*$^{-/-}$ cells. The arrow indicates ANKLE1 protein. f) Cell cycle profiles of HCT116 wild-type and *ANKLE1*$^{-/-}$ cells analyzed by FACS.

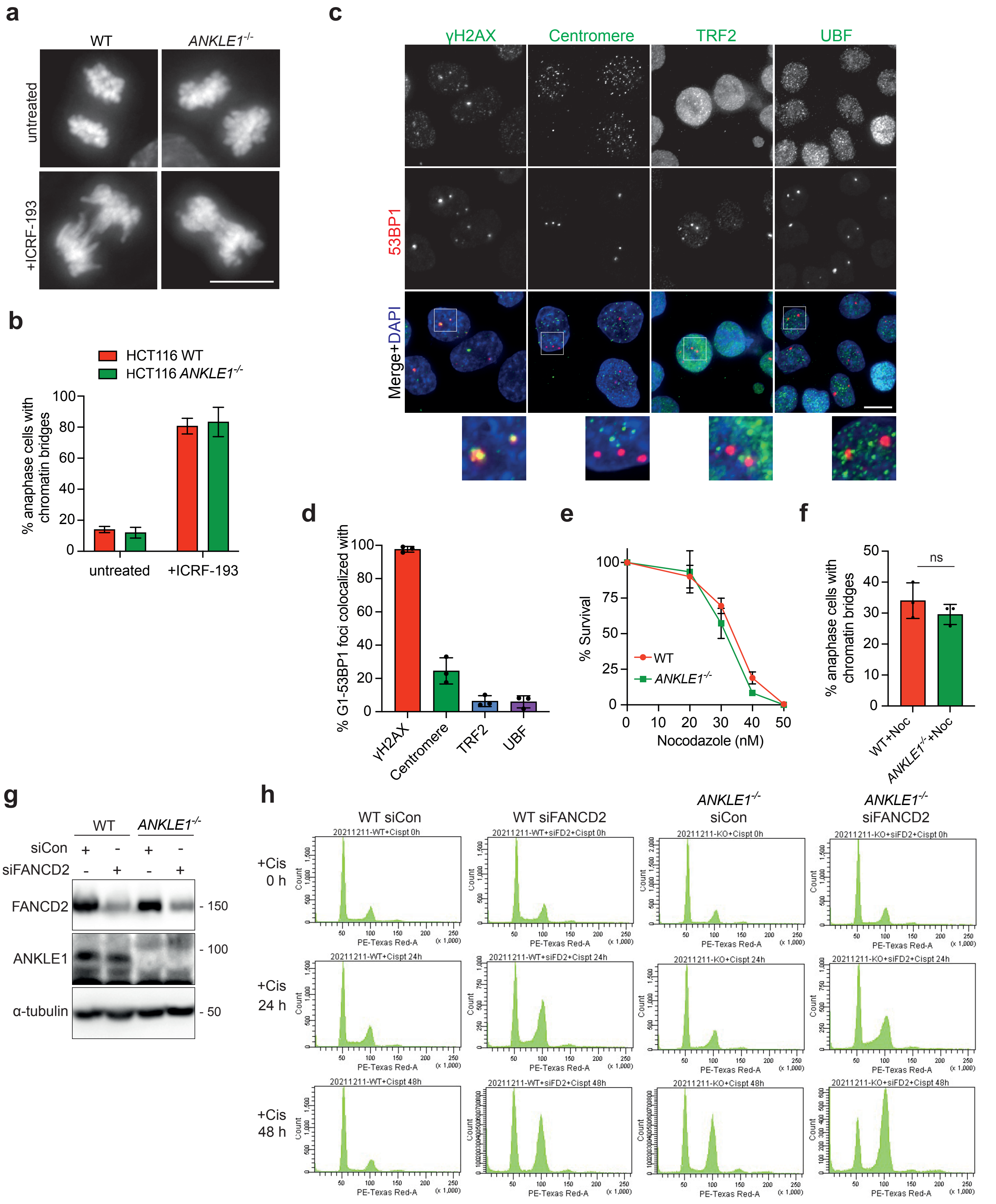


**Figure S3**. ANKLE1 and FANCD2 do not act in the same pathway. a) HCT116 and *ANKLE1*$^{-/-}$ cells were treated with or without ICRF-193 (100 nM) for 16 h and anaphase cells were imaged. b) Quantification of anaphase cells with chromatin bridges (> 150 cells per condition). c) γH2AX, centromere, TRF2, UBF (green), 53BP1 (red) and DNA (blue) were visualized in *ANKLE1*$^{-/-}$ cells. d) Quatification of colocalization between 53BP1 and γH2AX/centromere/TRF2/UBF in *ANKLE1*$^{-/-}$ cells (90 cells per condition). e) Clonogenic assay of WT and *ANKLE1*$^{-/-}$ cells treated wiht indicated concentrations of nocodazole. f) Quantification of nocodazole-treated anaphase cells with chromatin bridges (> 100 cells per condition). g) Cell extracts of cells treated with control siRNA or siRNA against FANCD2 were analyzed by western blotting for the indicated proteins. h) Cells were treated with control siRNA or siRNA against FANCD2. Cells were then treated with cisplatin (0.5 μg/mL) for the indicated time. Cell cycle profiles were analyzed by FACS. Scale bars, 10 μm. Bars represent mean ± SD of n = 3 independent experiments.

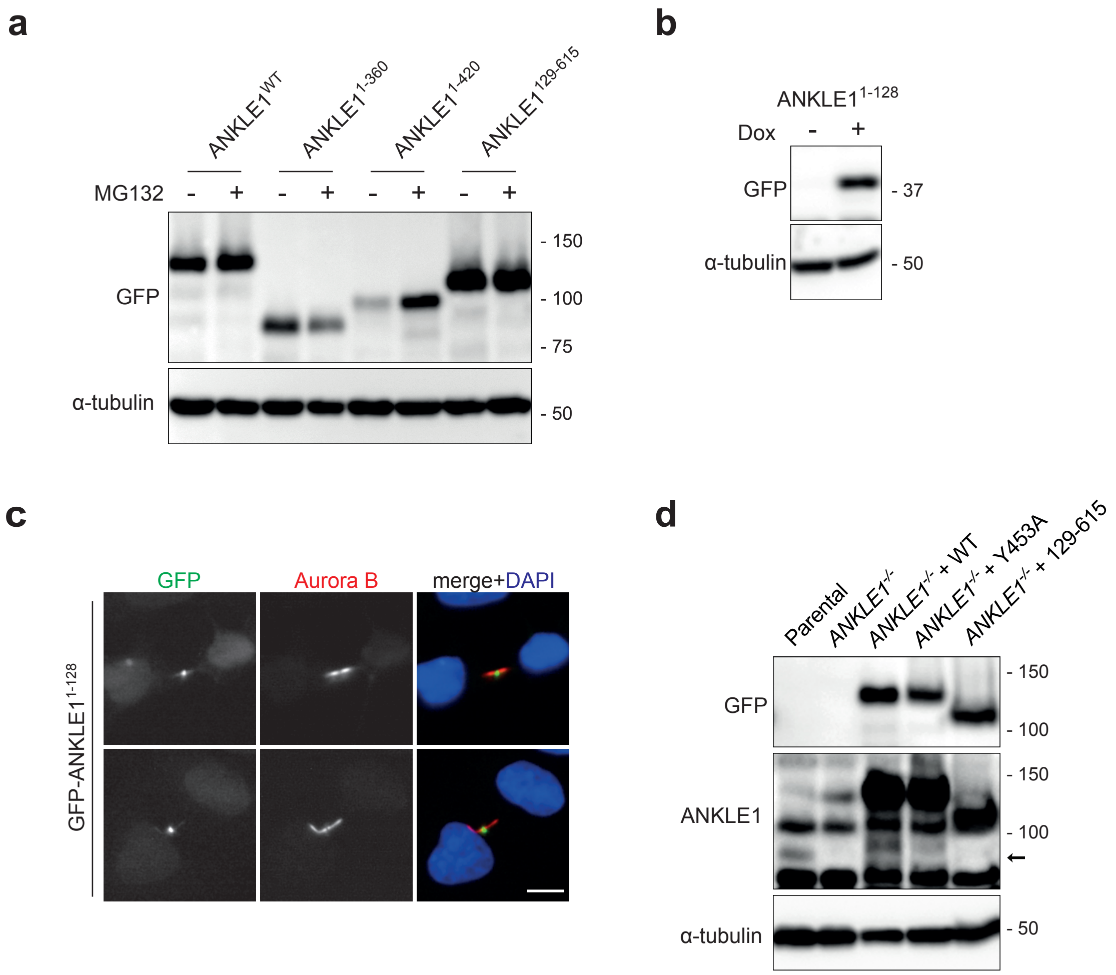


**Figure S4.** The N-terminal ankyrin repeats of ANKLE1 is sufficient to localize to the midbody. a) Cells expressing GFP-ANKLE1 constructs were treated with doxycycline (1 µg/mL) and with or without MG132 (10 µM). Cell extracts were analyzed by western blotting for the indicated proteins. b) Cells expressing GFP-ANKLE1$^{1\text{-}128}$ were treated with or without doxycycline (Dox). Cell extracts were analyzed by western blotting for the indicated proteins. c) Cells expressing GFP-ANKLE1$^{1\text{-}128}$ were treated Dox and were fixed for immunofluorescence. GFP (green), Aurora B (red) and DNA (blue) were visualized. Scale bar, 10 µm. d) Extracts of parental, *ANKLE1*$^{-/-}$, *ANKLE1*$^{-/-}$ cells rescued with the indicated ANKLE1 constructs were analyzed by western blotting for the indicated proteins. The arrow indicates endogenous ANKLE1 protein.

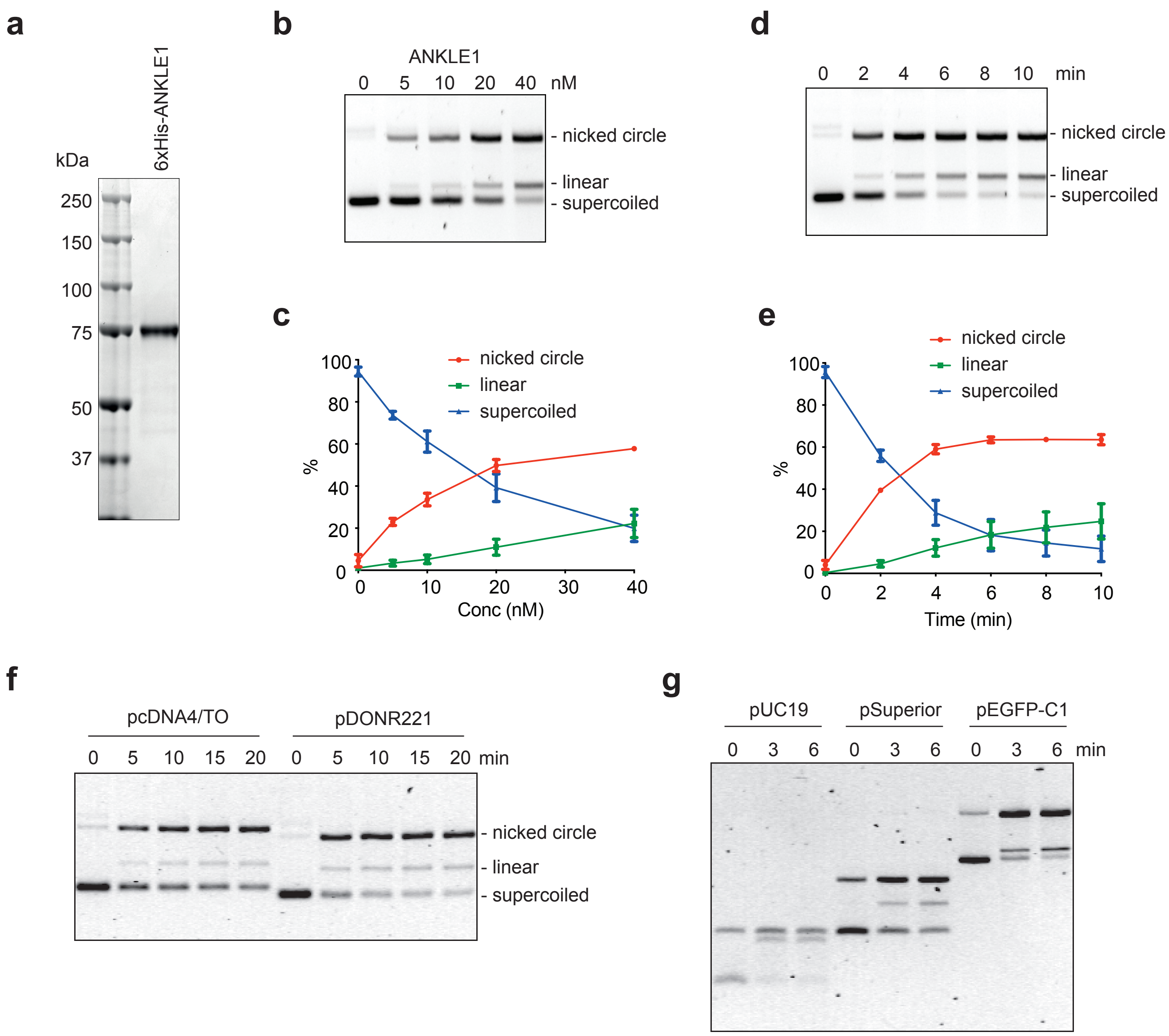


**Figure S5**. Cleavage of plasmid DNA by recombinant ANKLE1. a) Purification of full length 6xHis-ANKLE1 was analyzed by SDS-PAGE and stained with Coomassie blue. b) Plasmid pcDNA4/TO (0.5 nM) was incubated with the indicated concentrations of ANKLE1 for 5 min. Products were analyzed by agarose gel electrophoresis. c) Quantification of the DNA products, as determined in b). Graph shows mean ± SD from n = 3 independent experiments. d) Plasmid pcDNA4/TO (0.5 nM) was incubated with 20 nM of ANKLE1 for indicated time (0-10 min). Products were analyzed by agarose gel electrophoresis. e) Quantification of the DNA products, as determined in d). Plots show mean ± SD from n = 3 independent experiments. f) Cleavage of pcDNA4/TO and pDONR221 (0.5 nM) by ANKLE1 (10 nM). g) Cleavage of pUC19, superior and pEGFP-C1 (0.25 nM) by ANKLE1 (10 nM).

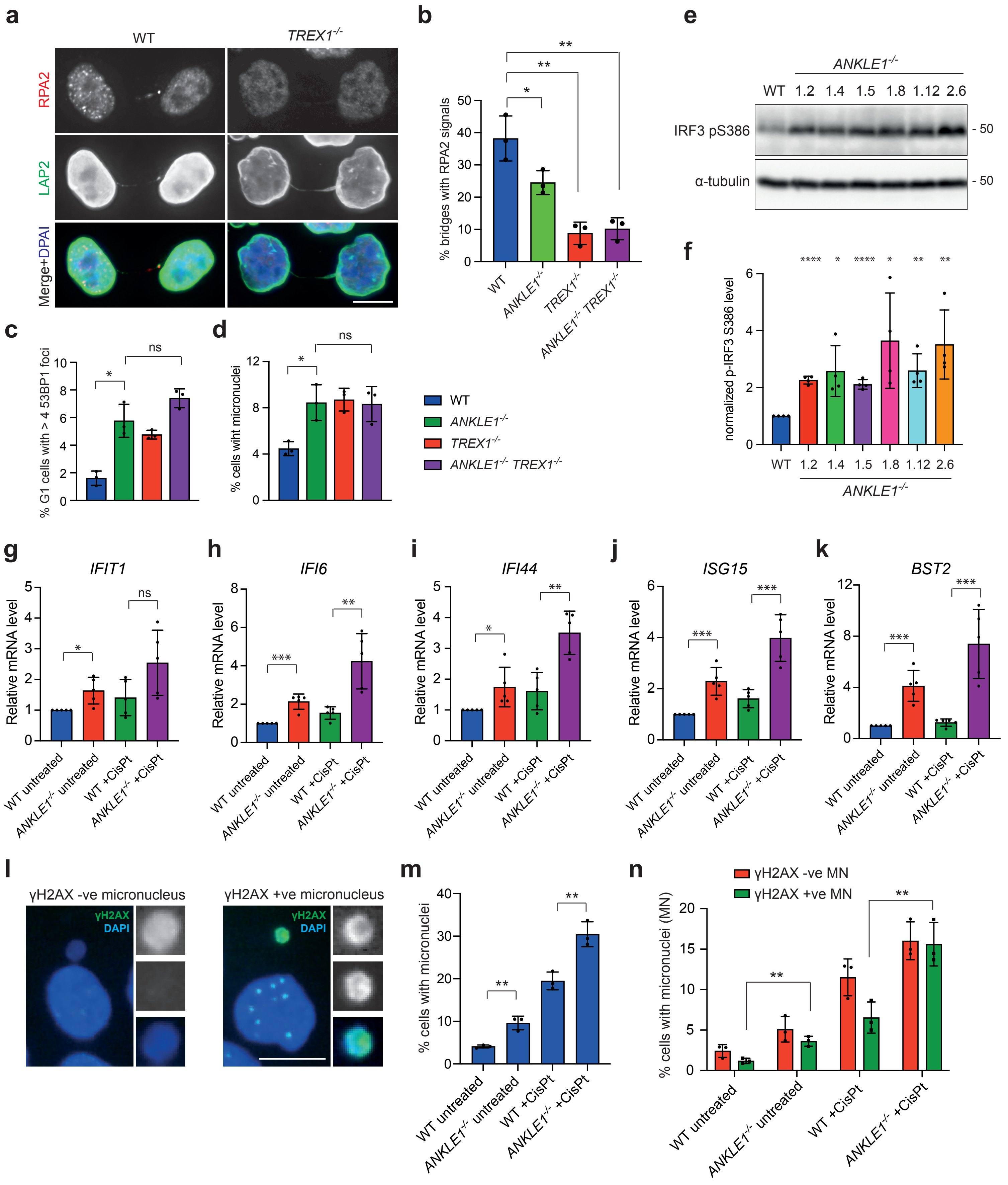


**Figure S6.** ANKLE1 deficiency promotes cGAS-STING activation. a) Representative images of wild-type and *TREX1*$^{-/-}$ cells with extended chromatin bridges. b) Quantification of chromatin bridges containing RPA2 as in a). Bars represent mean ± SD from n = 3 independent experiments. c) Quantification of cells (> 1000 cells per condition) with > 4 53BP1 foci. d) Quantification of cells (> 1000 cells per condition) with micronuclei. e) Cell extracts of wild-type and different clones of *ANKLE1*$^{-/-}$ cells were analyzed by western blotting for phosphorylation of IRF3 at S386. f) Quantification of the western blot signals of phosphor-IRF3 S386 normalized to tubulin from n = 4 independent experiments. g-k) Relative mRNA levels of the indicated ISGs in cells untreated or treated with cisplatin (0.5 μg/mL, 3 days) normalized to untreated HCT116 wild-type. Bars represent mean ± SD of n = 5 independent experiments. i) Representative images of cells showing γH2AX -ve or +ve micronucleus. m-n) Quantification of cells (> 3000 cells per condition) with micronuclei. Bars represent mean ± SD of n = 3 independent experiments. Scale bars, 10 μm.

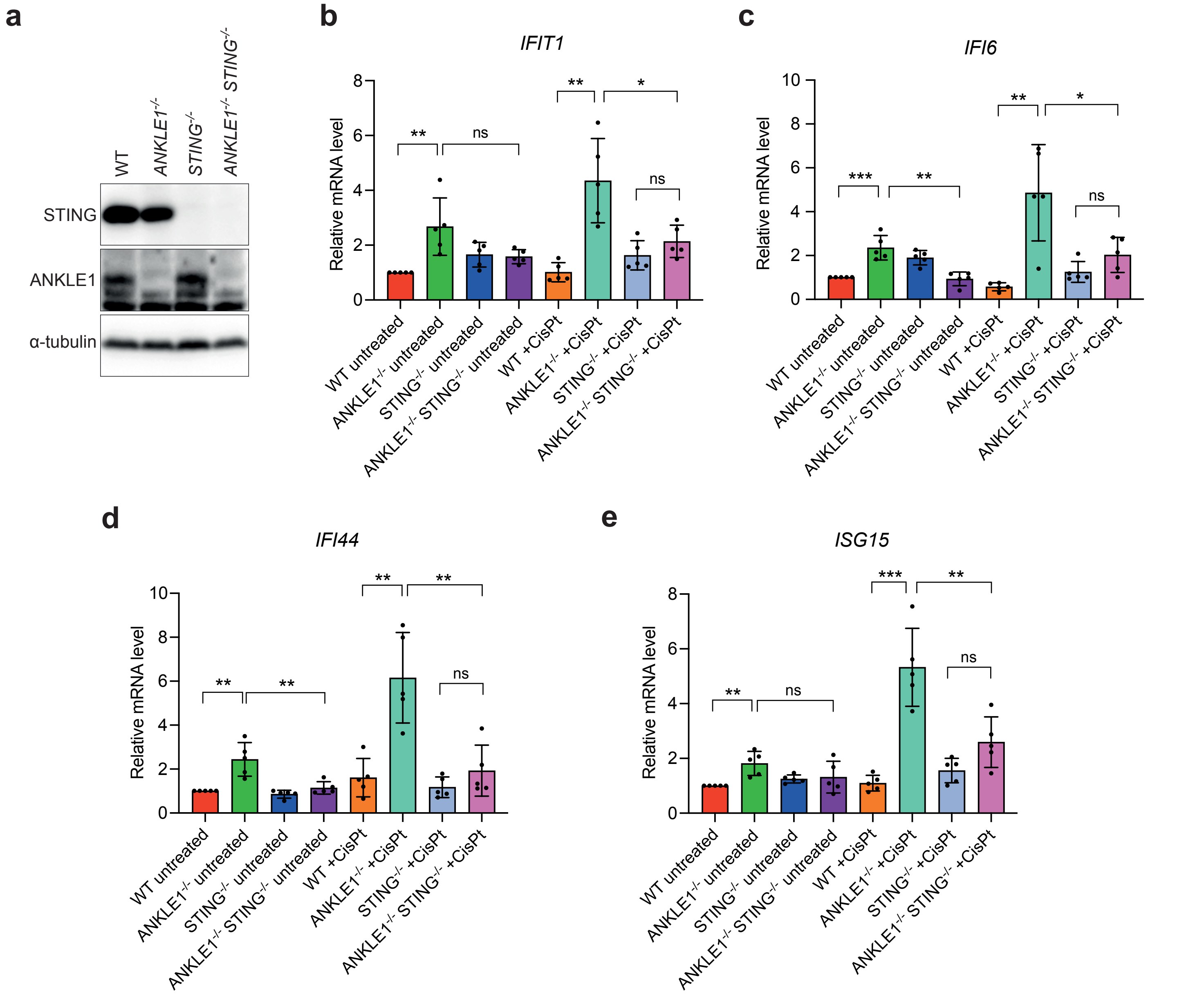


**Figure S7.** Induction of ISGs in *ANKLE1*$^{-/-}$ cells is dependent on STING. a) Cell extracts of the indicated cells were analyzed by western blotting. b-e) Relative mRNA levels of the indicated ISGs in cells untreated or treated with cisplatin (0.5 µg/mL, 3 days) normalized to untreated HCT116 wild-type. Bars represent mean ± SD of n = 5 independent experiments.

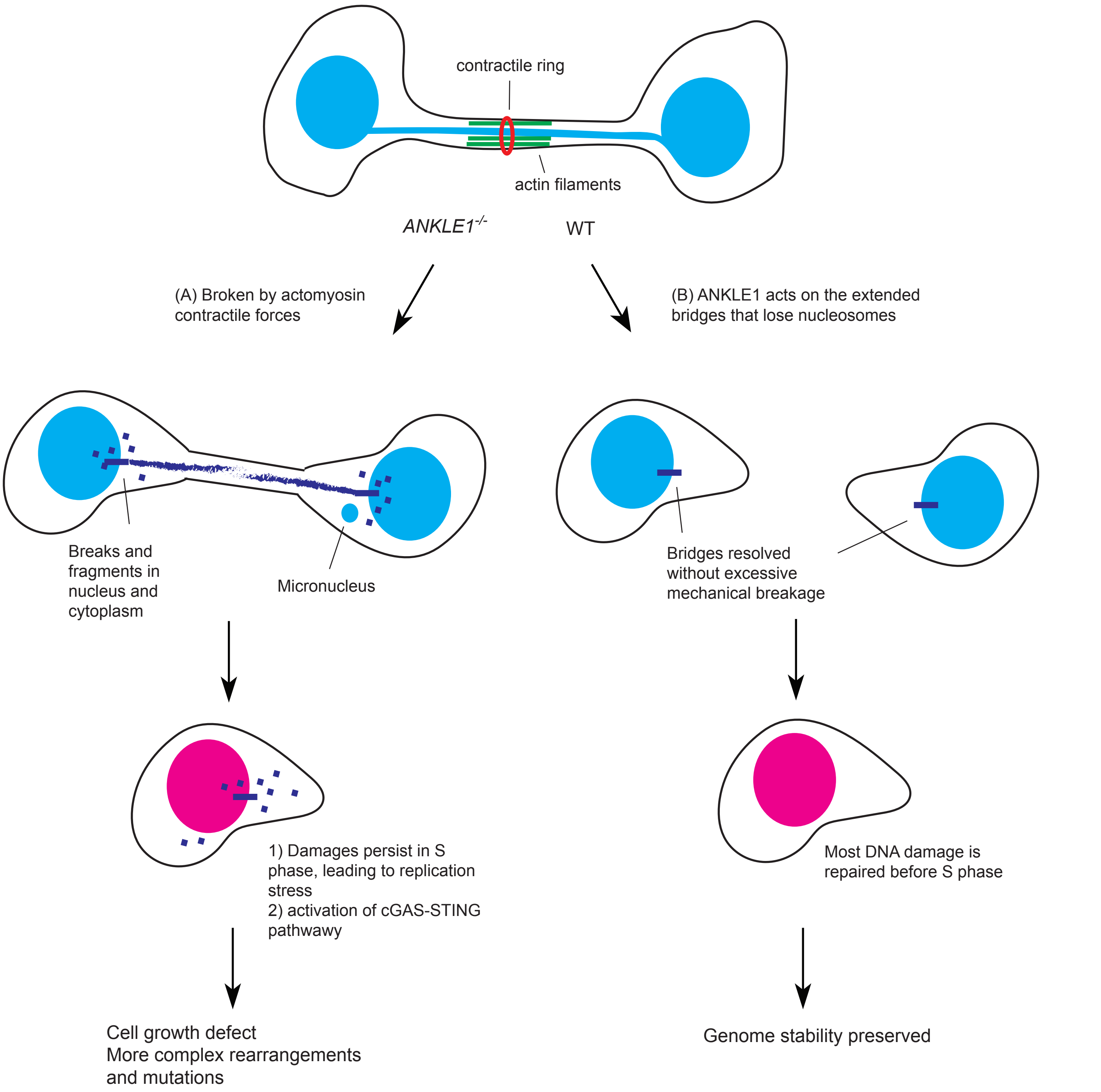


**Figure S8.** A schematic showing the cellular consequences when chromatin bridges are trapped at the cleavage plane in wild-type and *ANKLE1*$^{-/-}$ cells. See text for details.
